# Analysis and Practical Guideline of Constraint-Based Boolean Method in Genetic Network Inference

**DOI:** 10.1371/journal.pone.0030232

**Published:** 2012-01-17

**Authors:** Treenut Saithong, Somkid Bumee, Chalothorn Liamwirat, Asawin Meechai

**Affiliations:** 1 Bioinformatics and Systems Biology Program, School of Bioresources and Technology, King Mongkut's University of Technology Thonburi, Bangkok, Thailand; 2 Systems Biology and Bioinformatics Research Laboratory, Pilot Plant Development and Training Institute, King Mongkut's University of Technology Thonburi, Bangkok, Thailand; 3 Division of Biotechnology, School of Bioresources and Technology, King Mongkut's University of Technology Thonburi, Bangkok, Thailand; 4 Department of Chemical Engineering, Faculty of Engineering, King Mongkut's University of Technology Thonburi, Bangkok, Thailand; Niels Bohr Institute, Denmark

## Abstract

Boolean-based method, despite of its simplicity, would be a more attractive approach for inferring a network from high-throughput expression data if its effectiveness has not been limited by high false positive prediction. In this study, we explored factors that could simply be adjusted to improve the accuracy of inferring networks. Our work focused on the analysis of the effects of discretisation methods, biological constraints, and stringency of Boolean function assignment on the performance of Boolean network, including *accuracy*, *precision*, *specificity* and *sensitivity*, using three sets of microarray time-series data. The study showed that biological constraints have pivotal influence on the network performance over the other factors. It can reduce the variation in network performance resulting from the arbitrary selection of discretisation methods and stringency settings. We also presented the *master Boolean network* as an approach to establish the unique solution for Boolean analysis. The information acquired from the analysis was summarised and deployed as a general guideline for an efficient use of Boolean-based method in the network inference. In the end, we provided an example of the use of such a guideline in the study of *Arabidopsis* circadian clock genetic network from which much interesting biological information can be inferred.

## Introduction

Genetic network inference has been a widely studied research area since systems biology was introduced to unwind the complex regulation underlying biological systems. A genetic network which is a map describing relationships among genes is usually inferred from genomic information as well as genome-wide experimental data [1]. The experimental procedure for acquiring the genetic network has been reported [2].

Extensive studies of genetic network inference techniques have been reviewed by Bansal M *et.al.* [3]. The authors outlined the available computational analyses for converting gene expression profiles from microarray experiment to network, and highlighted the achievement of the reverse-engineering algorithms on such a work. Data from all kinds of molecular measurements are, in fact, useful for genetic network reconstruction and gap fulfilment; however, gene expression profiles are seemingly the most used data for which many network inferring techniques are developed. Literature of the network inference techniques indicates that the widely employed methods can be divided into four groups: (1) clustering-based (correlation of the gene expression; *e.g.* [4]), (2) Boolean-based (*e.g.* [5]), (3) Bayesian-based (*e.g.* [6], [7]), and (4) *in silico*-based (reverse-engineering; *e.g.* [3]) methods. Though the principles underlying each particular method are varied, these methods compute the relationship between genes based upon the similar hypothesis, *i.e.* genes are presumed to have relationship when their expressions are correlated. Clustering the similar pattern of gene expression profiles is a naïve computational method to identify the group of genes that share the transcriptional regulators in the network, but the prediction power of this method is limited at the cluster level and difficult to reach the individual gene pair level. This issue is taken by Boolean-based and Bayesian-based methods in which the relationship of each gene pair is calculated through logical and statistical theories, respectively. With a distinct goodness and limitation, Boolean-based and Bayesian-based methods are highly competitive in this research area. Both methods are of capable to analyse large-scale data, yet under the critical concerns of computational burden for Bayesian-based method and a high false positive rate for Boolean-based method. In contrast with the aforementioned groups whose analyses rely on the experimentally measured data, reverse engineering algorithm which is facultative as the *in silico*-based method carry out the computation based upon the in *silico* dataset given by the model. It is, in principle, an exploitation of the model simulation to generate the data of interest for the analysis. This method may be advantageous only for the relatively small scale datasets, because simulation of a large system is technically not easy and often faces computationally infeasible situation [3], [8].

Compared among the existing methods for network inference, Boolean-based analysis is relatively a simple method that is applicable to large-scale data analysis. The accuracy of the Boolean network may not be as high as that of the Bayesian network, yet it is in compensation with its simplicity and computational usage. The modifications applied to Boolean network are driven by the attempts to utilise its advantages in diverse applications of network inference. Originally, Boolean network relies only on a simple logical theory of a binary system, *i.e.* “on and off”, “0 and 1”, or “true and false” [9], [10]. About ten year ago, such a Boolean network was introduced to the genetic network inference application, for example inferring the genetic network of cell cycle process using gene expression data [5]. Later, Probabilistic Boolean network was initiated to improve the accuracy of the Boolean approach. All possible networks are generated according to a family of Boolean functions, among which the network giving highest statistical score is presumed to be a final inferred network [11]. In an extent to support the time-series data, Martin and colleagues have provided a Boolean-based method so called “Boolean dynamics”, whereby the inferred network is selected from the possible Boolean networks using steady-state dynamics [1]. Furthermore, Bumee *et al* recently introduced a constraint-based Boolean method that strategically exploits prior knowledge as biological constraints to reduce the false positives in the inferring network [12].

Extensions of Boolean network are most likely performed to serve a specific application rather than an attempt to resolve the weakness of the method itself. As reviewed, Boolean network has been either modified to support various types of data or extended by *ad hoc* theories. Very limited research has addressed such weaknesses in high false positive rate and dependency of discretisation methods of the classical Boolean network. This study hence investigated the effect of discretisation methods and biological constraints from prior knowledge on the overall performance of Boolean network, including the reduction of false positive prediction. Furthermore, we questioned whether adjusting the stringency of Boolean function assignment, which herein refers to the restriction of the function assignment against the conflict of the Boolean function occurring within the a time-series profile, can improve the performance of the Boolean network. The complete analysis was performed using three time-series datasets having distinct characteristics in terms of quality (amplitude of the expression) and resolution (number of datapoints): (1) galactose system – seven datapoints with low amplitude profiles [13], (2) circadian clock under constant light condition (LL) – twelve datapoints with higher amplitude profiles [14], and circadian clock under light/dark condition (LD) – six datapoints with highest amplitude profiles [15]. The results of the analysis were summarised and established as a guideline for facilitating the application of Boolean-based analysis to the genetic network inference. Finally, we demonstrated the use of the proposed guideline in the inference of a circadian clock network.

## Methods

**Figure 1:**
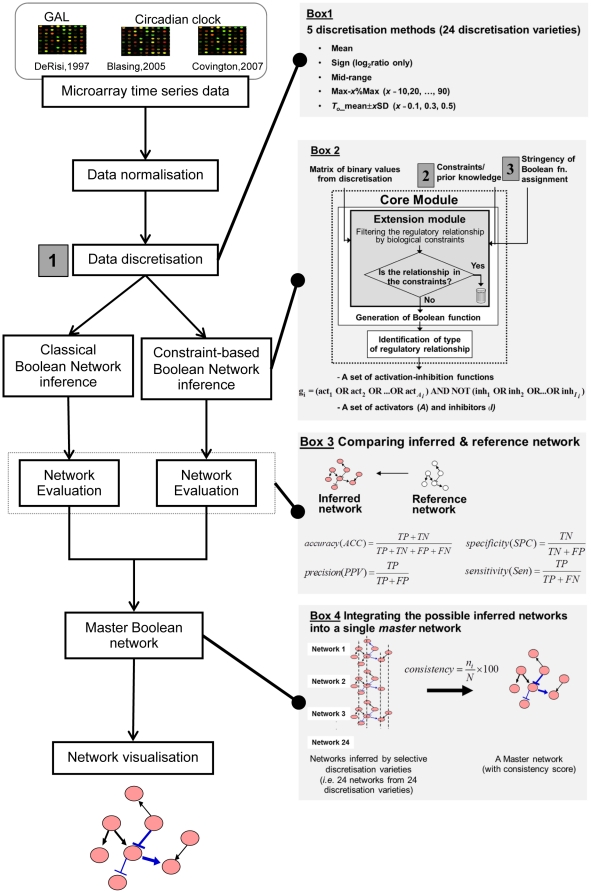
Overall methodology. In this study, the methods can be divided into 4 parts described in the Boxes 1–4; Box 1 – discretisation methods, Box 2 - constraint-based Boolean method, Box 3 – network evaluation, and Box 4 – master network concept.

This work aims to study the influence of discretisation methods (labelled as 1 in Figure 1; see Box 1), biological constraints (labelled as 2 in Figure 1; see Box 2), and stringency level of Boolean function assignment (labelled as 3 in Figure 1; see Box 3) on the correctness of the inferred genetic networks with respect to the reference network (see Box 3). The correctness of the network that indicates the predictive power of the Boolean-based method with a certain setting was evaluated through the network performance indexes, consisting of *accuracy*, *precision*, *specificity* and *sensitivity*. Then, the multiple network solutions resulting from a variety of the selected discretisation methods were integrated through a concept of master network (see Box4) in which the final network was passed to the visualisation protocol of Cytoscape [16]. Figure 1 shows the scheme of the overall methodology employed throughout the study. Our study relied on the analysis of three independent microarray time-series datasets possessing distinct characteristics [13], [14], [15].

### 1. Datasets

Three microarray time-series datasets studied herein consist of one cDNA microarray dataset of galactose utilisation pathway in *Saccharomyces cerevisiae* during diauxic shift from fermentation to respiration (denoted as galactose system) [13] and two Affymetrix microarray datasets of circadian clock in *Arabidopsis thaliana* under different light exposure (denoted as circadian clock system) [14], [15]. The former dataset was downloaded from http://cmgm.stanford.edu/pbrown/explore/, where the full description of the data was found. In brief, it is seven-datapoint time-series data exhibiting the response of galactose utilisation pathway to the decline of glucose concentration (19, 18.7, 17.6, 14, 7.5, 0.2, and 0 g/l). For the latter two datasets, the Affymetrix CEL files were downloaded from NCBI database (http://www.ncbi.nlm.nih.gov; experiment reference numbers are GSE3416 and GSE8365): twelve-datapoint time-series data is a measurement of circadian clock under continuous light (LL) condition [14], while six-datapoint time-series data is a measurement of such system under light/dark cycle (LD) condition [15].

By using the well-known biological systems as case studies, it is advantageous to have abundance of evidence to reduce dimension of considering data. Herein, a certain number of relevant and irrelevant genes to the pathways of interest were selected for being a test set to: (1) avoid complexity due to the extensive amount of data impeding our analysis, and (2) easily investigate the prediction power of the protocol. For galactose utilisation pathway, twenty genes were selected from the original dataset, consisting of seventeen relevant genes (*GAL1*, *GAL2*, *GAL3*, *GAL4*, *GAL7*, *GAL10*, *GAL11*, *GAL80*, *PGM1*, *PGM2*, *LAP3*, *EGD1*, *EGD2*, *MIG1*, *SSN6*, *TUP1*, and *SNF1*) and three irrelevant genes (transcription factors in cell cycle; *TFB1*, *TFB2*, and *TFB3*) [17], [18]. For the circadian clock system, the identical fourteen genes were chosen from both conditions [19], including seven relevant genes in circadian clock (*CCA1*, *LHY*, *TOC1*, *GI*, *PRR5*, *PRR7*, *ELF4*) and the other seven genes in glycolysis and flowering pathways (*CO*, *FT*, *SOC1*, *TPI*, *PGM*, *PGK*, *PGI1*).

### 2. Data normalisation

The measured intensity of cDNA microarray contains both foreground (red (*R_f_*) and green (*G_f_*)) and background (red (*R_bg_*) and green (*G_bg_*)), so that the differentiation of gene expression between conditions is evaluated through the log_2_ratio (log_2_(*R_f_*−*R_bg_*/*G_f_*−*G_bg_*)) of the spot intensity after background subtraction [20]. For Affymetrix microarray data, the normalisation was based on the standard procedure deployed by Bioconductor package (http://www.bioconductor.org). Briefly, CEL files of the two datasets were normalised to account the variation of arrays by RMA-quantile [21], [22] and qspline methods [23]. The probe-specific background subtraction was relied on PM-only method, while Affymetrix chip correction was done through Li-Wong invariant set method [24].

### 3. Data discretisation

Discretisation is a process to encode expression data into binary values [25], [26]. Five different discretisation methods were studied. The differences among the methods lie on different criteria for discriminating the binary state of the data as described below. Let *T* = {*T_0_, T_1_, T_2_, …, T_i_, …, T_n_*} are expression values measured along the time series *t* = {*t_0_, t_1_, t_2_ , …, t_i_, …, t_n_*}; and *DT* = {*DT_0_, DT_1_, DT_2_, …, DT_i_, …, DT_n_*} are series of the discretised values in correspondent with the expression values *T*.

- **Mean.** Mean was referred to the arithmetic average of expression across time points. For this method, all expression values that are greater than the mean were encoded to 1, and 0 otherwise (Equation 1).
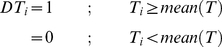
(1)
- **Mid-range.** Mid-range was referred to the centre point of the distance between the maximum and minimum of the expression profile. All expression values that lie above the mid-range were assigned as 1, and 0 otherwise (Equation 2).
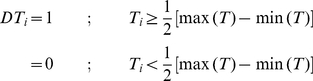
(2)
- **Sign of log_2_ratio.** Taking log_2_ratio of the expression data resulted in a set of real number which can be discretised through the sign. All positive real numbers were encoded to 1, and 0 otherwise. This method is limited to the discretisation of log-ratio type of data.
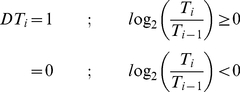
(3)
- **Max - x%max.** Max – *x*%max was referred to the value relative to the highest point across the expression profile. *x* can range from 0 to 100 percent. However, for this study we varied the percent of *x* from 10 to 90 with 10 percent interval. All expression values that are greater than the max - *x*%max were encoded to 1, and 0 otherwise (Equation 4).
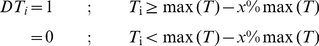
(4)
- **T_o__mean ± xSD.** Unlike the above methods, the criteria for discretisation of this group were rather complicated. Mean was also employed in the value discrimination, yet there are two major differences from the first method: (1) the flexibility of the criteria was applied in terms of fold of standard deviation (*x*SD; where *x* = 0, …, 1) and (2) the values were discretised in accordance with the state given to the initial time point in the series (*T_o_*), which was encoded into 0 or 1 based on the mean (mean *T_o_*), maximum (max *T_o_*), or minimum (min *T_o_*) expression value of an expression profile. In this work, *x* was varied as 0, 0.1, 0.3, or 0.5. The expression values that are greater than mean + *x*SD were encoded to 1, while the expression values that are less than mean − *x*SD were encoded to 0. The expression values that lie between the mean ± *x*SD thresholds were encoded to the same state as its preceding state (Equation 5).
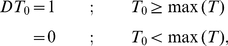
(5)

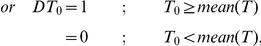


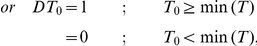


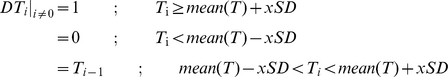

By varying the parameter settings for max - *x%*max (*i.e. x*) and *T_o_* _mean ± *x*SD (*i.e. T_o_* and *x*), in total we have 24 discretisation varieties for our study: mean, mid-range, sign of log_2_ratio, max-*x*%max (9 varieties from *x* = 10 … 90), and *T_o_* _mean ± *x*SD (12 varieties from min *T_o_*, mean *T_o_*, max *T_o_* and *x* is {0, 0.1, 0.3, 0.5}). The goodness of each discretisation method was assessed through the inferred network performance (described in section 5 of Methods).

### 4. Boolean-based methods

#### Classical Boolean network

For the classical Boolean network [1], microarray time-series data were analysed without consideration of any prior knowledge. The regulators were identified based on Boolean function under a hypothesis of heuristic regulation that the expression of the target gene is a consequence of the expression of the regulator gene with a certain delay. The Boolean function, thus, represents the states of regulation regarding the regulatory control in the genetic network.

#### Constraint-based Boolean network

The constraint-based Boolean network [12], an extent of the classical Boolean [1], provides an option to integrate the prior knowledge, which could be either the well-known fact or the background information regarding the biology of the system, into the analysis regime. In the context of our study, the constraint-based Boolean network that was originally presented by [12] was employed to investigate the influence of the prior biological knowledge on the network performance. Briefly, the biological knowledge was incorporated into the analysis regime as constraints for Boolean function assignment. For the sake of simplicity, the regulation inferred was restricted to the transcriptional level excluding self-regulation, so that the post-transcriptional regulation, *e.g.* the feedback control from metabolic enzymes was beyond the scope of the method. Specifically, an enzymatic gene was not allowed to be a parental node in the Boolean analysis (so called the “*conceptual constraints*”). Moreover, the known non-regulated gene-pairs were exploited in the framework as *“specific constraints”*. The example of the advantage of such constraints and the further information on how they can be implemented can be found elsewhere [12].

For Boolean function assignment, the level of stringency was precisely given, instead of following the *ad hoc* majority rule. The level of stringency, in other words, indicates the threshold of the conflicted allowance in the process of Boolean function defining. Herein, the level of stringency was set at 50, 80, and 100 percent corresponding to the conflicted allowance of the Boolean function assigned across time-points at 50, 20, and 0 percent, respectively.

### 5. Network evaluation

The inferred genetic network from each discretisation variety was assessed using network performance indexes, including *accuracy*, *precision*, *sensitivity*, and *specificity*. Through the comparison of gene-gene relationships comprised of the network, these performance indexes indicate the correctness of the inferred network based upon the matches between the inference and reference networks. This assessment method was used according to the presumption that the network that possesses similar components to the realism (*i.e.* reference network) contains similar behaviour. *Accuracy* is the proportion of the correct prediction (T) against all predicted results. *Precision* is the proportion of true positive (TP) against all positive predictions (P). *Specificity* is the performance of negative identification, while *sensitivity* is the performance of positive identification. The equations of the network performance indexes are given in Equations (6)–(9):
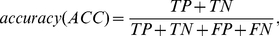
(6)

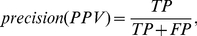
(7)

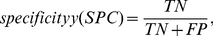
(8)

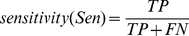
(9)where *TP* (true positive) refers to the number of relationships correctly inferred, *FP* (false positive) refers to the number of false predicted relationship, consisting of prediction of unidentified data and wrong prediction, *TN* (true negative) refers to the number of non-relationships correctly inferred, and *FN* (false negative) refers to the number of existing relationships that cannot be inferred. The reference networks to which the predictive networks were compared for identifying *TP*, *TN*, *FP*, and *FN* were concluded from the literature as schematised in Figure S1B: (1) for galactose [13], [18] and (2) for circadian clock [19] systems. These reference networks are presumed to be a true regulation occurring inside living organisms, so that they function as the true networks in the calculation of the performance indexes.

### 6. Determination of the effect of data quality and resolution on the inferred network

The quality of the data can be evaluated through many data characteristics; however, this work was focused on the aspect of the clarity in data pattern. At the particular study of oscillatory time-series data, amplitude was employed to reflect the quality of data. In the context of microarray time-series data containing *p* datapoints of *k* genes, the amplitude of the dataset is given by
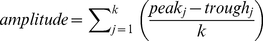
(10)
*peak_j_* and *trough_j_* are the highest and lowest expression values of the *j^th^* gene, respectively. Apart from the amplitude-based quality, the number of datapoints (*p*) in a time-series profile was used to represent the resolution of the dataset. The effects of both data quality and resolution were investigated through the plots of these quantities against the performance of the corresponding inferred networks.

### 7. Master Boolean network

For Boolean-based analysis of a single expression dataset, the series of networks resulting from a choice of discretisation methods and settings were summarised into one network solution called *master Boolean network*. The gene relationships comprised of such network were identified at least once in the networks inferred by a variety of Boolean settings. The consistency of the inferred gene relationships among the series of network solutions (Equation 11) indicates the confidence of such relationship. The consistency is subsequently exploited as a filter criterion for discarding the relationship that could be found by chance. As for our study, the *master Boolean network*s were plotted from the gene relationships that possess at least 20 percent consistency so as to simplify the resulting network.
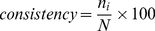
(11)
*n_i_* denotes the number of network solutions that contains the relationship *i* from the total *N* solutions.

## Results

### 1. Analysis of the Boolean-based method for network inference

**Figure 2:**
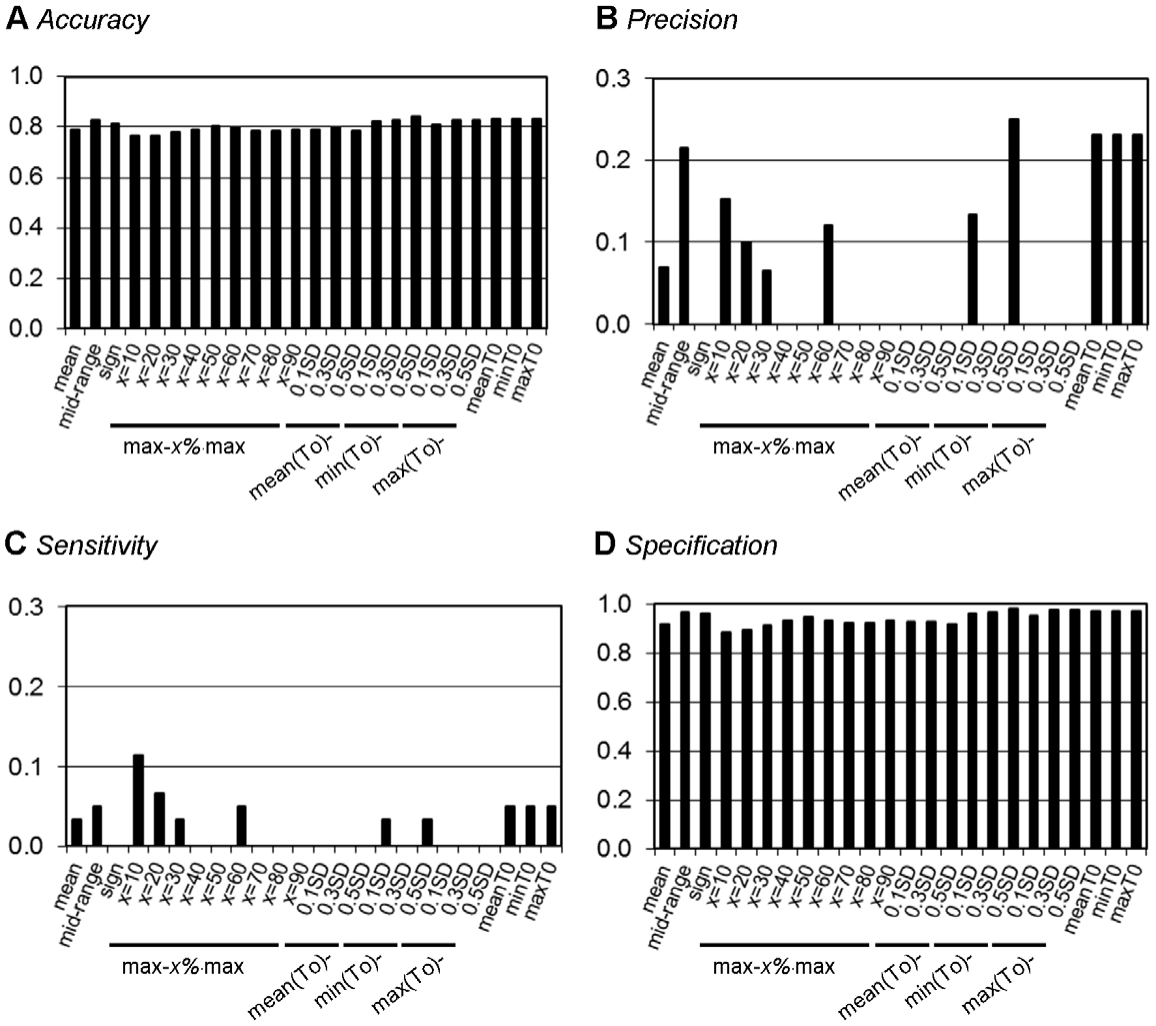
Evaluation of Boolean network performance for Galactose system (data from [**13**]). The results were obtained from the constraint-based Boolean network slightly modified from [12]: (A) accuracy; (B) precision; (C) sensitivity; (D) specificity.

**Figure 3:**
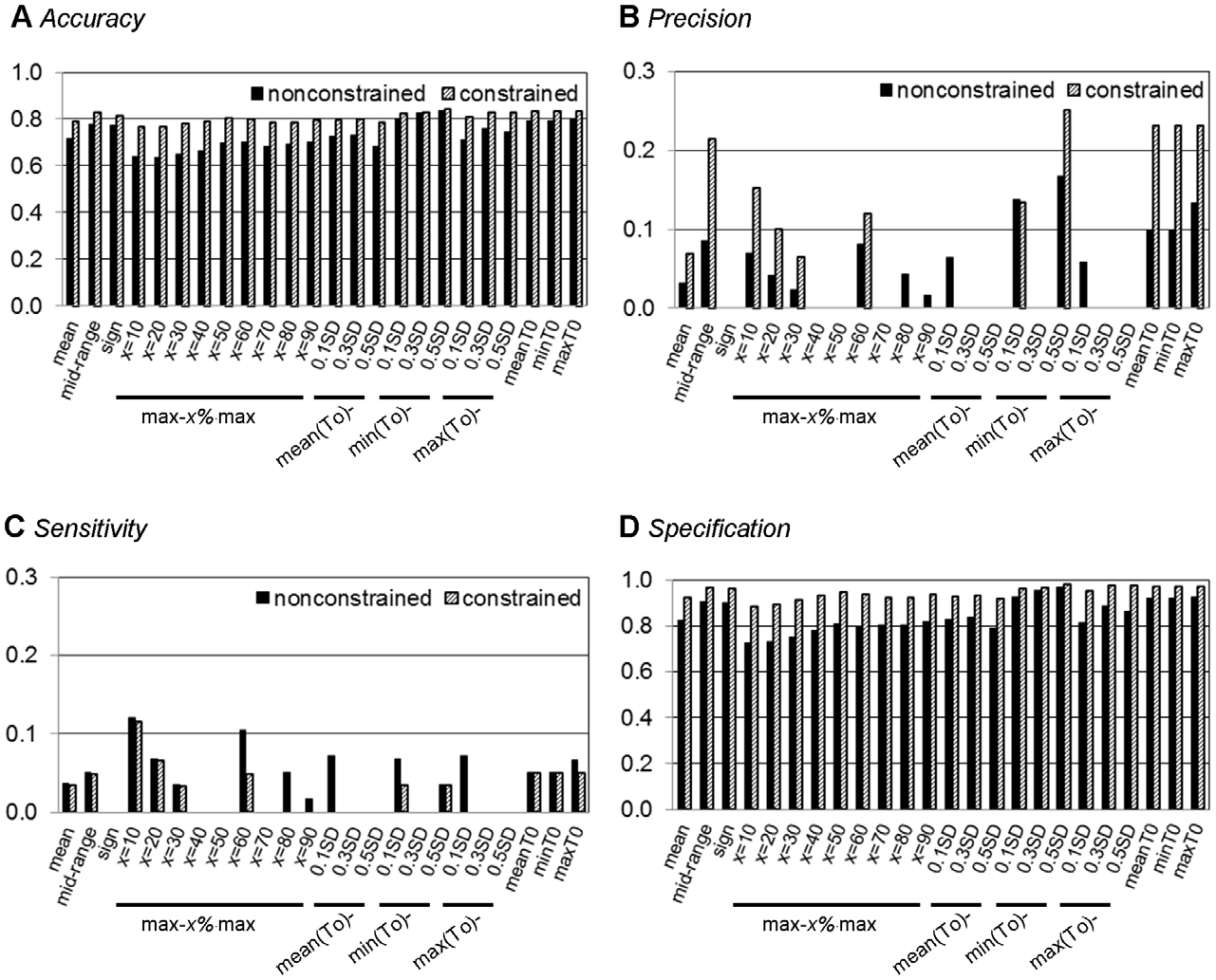
The effect of constraints on the Boolean network inference regime. The performances of constraint-based Boolean network for Galactose system (data from [13]) were compared with those of the classical Boolean network inference: (A) accuracy; (B) precision; (C) sensitivity; (D) specificity; and black – non-constrained; hatch – constrained).

**Figure 4:**
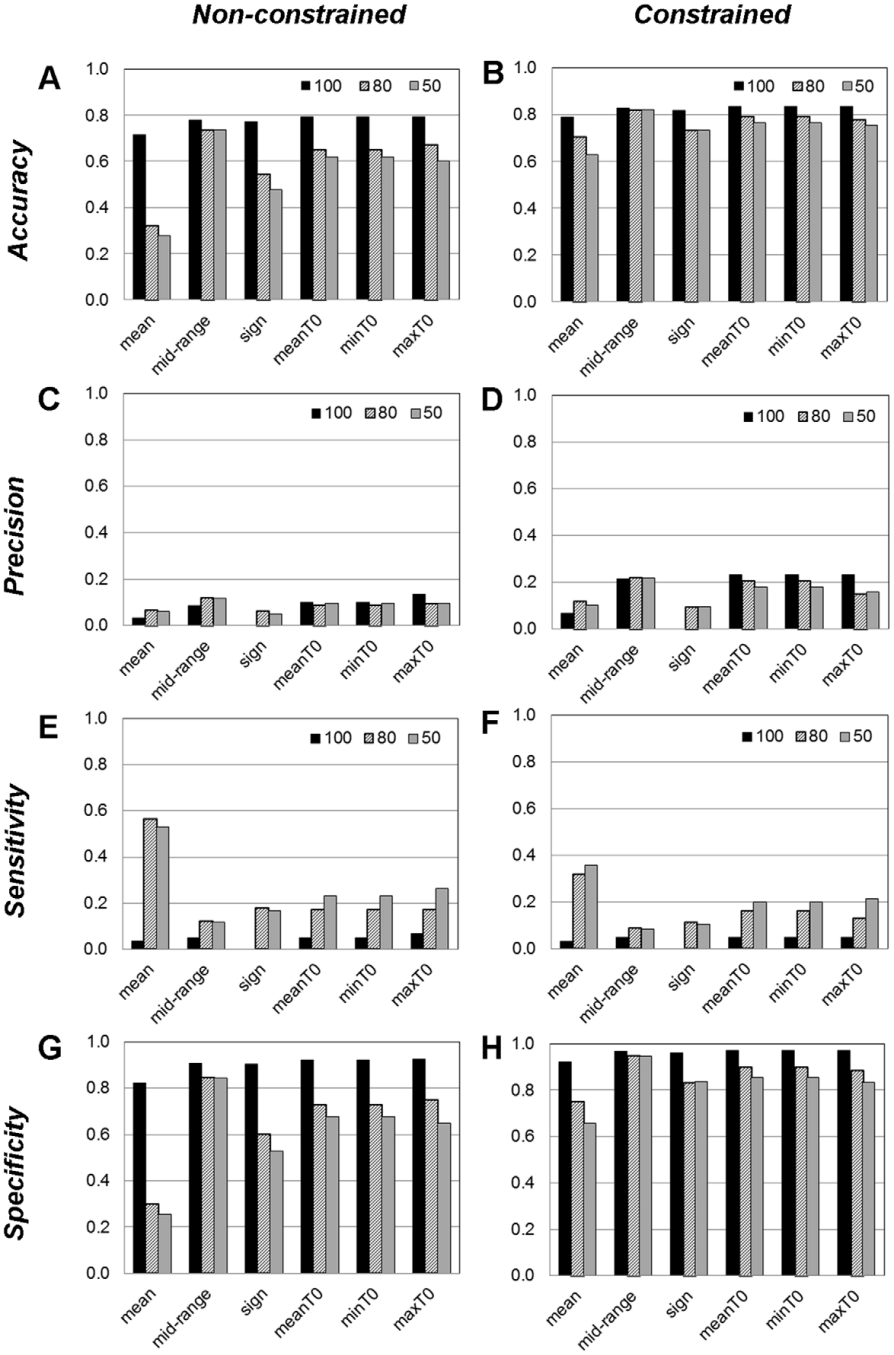
The effect of level of stringency of Boolean function assignment on the Boolean network inference regime. The performances of constraint-based Boolean network for Galactose system (data from [13]) (right; A,C,E,G) were compared with those of the classical Boolean network (left; B,D,F,H) inference under different levels of stringency of Boolean function assignment: black – 100, hatch – 80, and grey – 50 percent).

This work investigated the influence of the factors determining the performance of the Boolean network, focusing on discretisation methods, biological constraints, and level of stringency of Boolean function assignment. The study was performed based on three sets of microarray time-series data to avoid the bias on a selective dataset (Figure S1A): galactose systems [13], circadian clock in LL [14] and in LD [15]. The results of galactose system were demonstrated to describe the findings of our analysis (Figures 2, 3, and 4), while the corresponding results of the other systems supporting such conclusions were reported in Figures S2, S3, S5, and S6.

#### Influence of discretisation methods

Discretisation is an essential step transforming time-series data into the discreted form of [25] which is the requirement of the Boolean function analysis. As a matter of fact that the inferred network varies with the employed discretisation methods [27], we exhibited such effect by investigating the performance of 24 networks of using 24 discretisation varieties derived from the five typical discretisation methods: mean, mid-range, sign of log_2_ratio, max-*x*%max (9 varieties from *x* = 10 … 90), and *T_o_*_mean±*x*SD (12 varieties from min*T_o_*, mean*T_o_*, max*T_o_* and *x* is {0, 0.1, 0.3, 0.5}). Previously, similarity score which measures the likeness of the discretised profile and the raw data was employed to evaluate the goodness of the method [28]. However, we proved here that the method giving the most likely discretised profile may not bring to the best inferred network (Table S1). In our study, the constraint-based Boolean networks of the different discretised data were therefore compared through four network performance indexes consisting of *accuracy*, *precision*, *sensitivity*, and *specificity* (see definition in Methods; Figures 2, S2, and S3). The results show that using different discretisation methods obviously affects the *precision* and *sensitivity* of the inferred networks, while their effect on the network *accuracy* and *specificity* is inferior. Within the scope of the study, we found that the mean, mid-range and *T_o_*_mean±*x*SD (at *x* = 0) always give generally good and comparable network performance in contrast with the max-*x*%max whose network performance is highly dependent on *x*. The results, thus, suggested that the mean, mid-range and *T_o_*_mean±*x*SD (at *x* = 0) could be considered as *ad hoc* discretisation methods for Boolean network inference.

#### Effect of biological constraints

Constraints derived from prior knowledge are often naively incorporated into Boolean analysis to reduce the complexity of resulting networks. An incorporation of the constraints into Boolean analysis has been previously reported to improve accuracy of the inferred network [12], [29]. Our study used the independent datasets to support the impact of constraints on the improved network performance (Figures 3, S2, and S3). We found that the constraint-based Boolean regime enhances the *accuracy*, *precision*, and *specificity*, but not *sensitivity*, of the inferred network when compared with those of the classical Boolean approach. We also showed that the better network performance is a consequence of the reduction of false positive (FP) prediction and the elevation of true negative (TN) prediction (Figure S4). In addition to the improvement of the *accuracy*, *precision*, and *specificity* of the inferred network, the constraints can reduce the dependency of the inferred network on the discretisation methods (Figures 3, S2, and S3).

#### Effect of the level of stringency for Boolean function assignment

The stringency of Boolean function assignment is another parameter embedded in Boolean analysis regime that could determine the performance of the inferred network, especially for time-series data. In this study, we examined whether relaxing the stringency of Boolean function assignment enables the improvement of the network performance. Using the discretisation methods that give a generally good result in the previous test (*i.e.* the mean, mid-range, sign of log_2_ratio and *T_o_*_mean±*x*SD (at *x* = 0)), the level of stringency for the (constraint-based) Boolean analysis was subjectively declined from 100 to 50 percent. Figure 4 demonstrates the performance of the networks for the galactose system inferred by Boolean analysis regimes both with (right panel) and without biological constraints (left panel). Similar results were observed in both regimes that neither network performance indexes, except *sensitivity*, were improved by relaxing the stringency level of Boolean function assignment. Particularly for the constraint-based regime, the level of the stringency seems to have less influential on the inferred network. The consistent results were also found in the evaluation of the network performance of the circadian clock systems (Figures S5 and S6).

**Figure 5:**
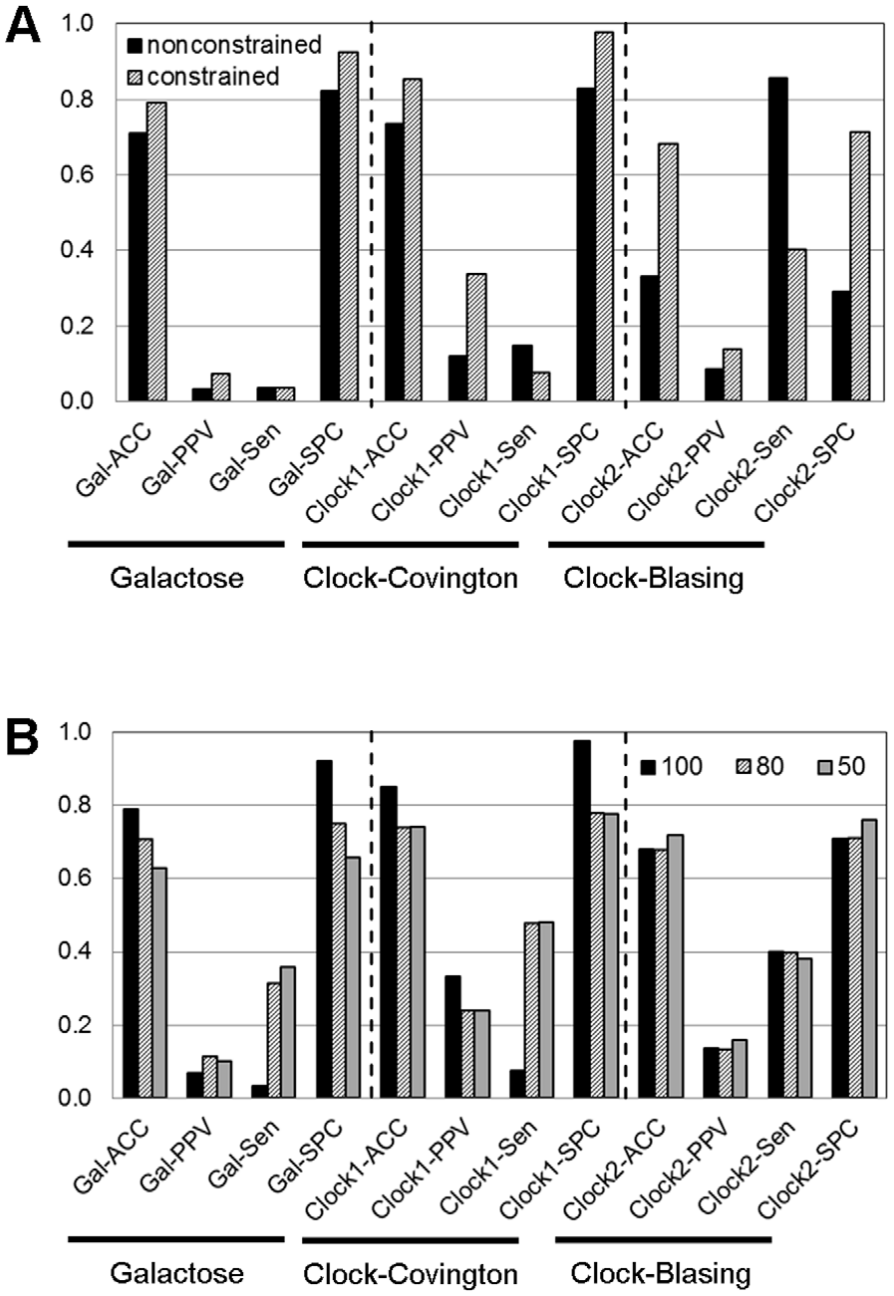
The effect of constraints and stringency on the Boolean network inference regime among the observed datasets. The performances of Boolean networks were evaluated and compared under the (A) constrained and (B) varied stringency conditions. The studied datasets included (1) Galactose system (Gal: data from [13]), (2) circadian clock system under constant light (Clock1: data from [14]), and (3) circadian clock system under light/dark cycle (Clock2: data from [15]).

To ensure that our findings are not dataset-specific results, a quick comparison of the analysis results among the three datasets was provided in Figure 5, in which the performance of the inferred networks (based on mean discretisation) for the galactose and circadian clock systems were plotted side-by-side: (A) for contribution of the constraints and (B) for the relaxation of stringency of Boolean function assignment. It is worth noting that the time-series data (Figure S1A) chosen for this study contain a variety in characteristics based upon the amplitude of the profile (inferring *data quality*) and the number of datapoints (inferring *data resolution*). The consistency of the results from these three datasets (as observed in Figure 5) thus indicates the generality of the findings in this work.

#### Effect of data quality and resolution

**Figure 6:**
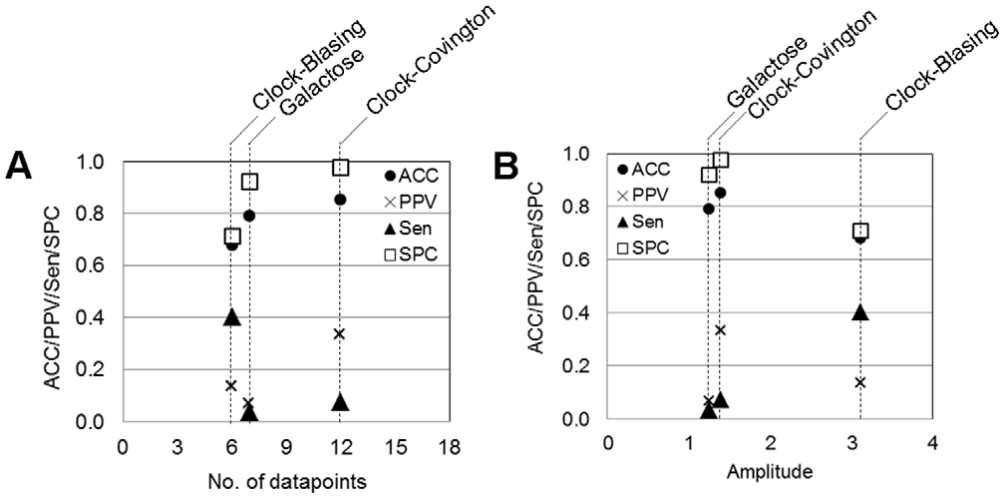
The effect of the number of (A) data-points and (B) amplitude on Boolean network inference. The constraint-based Boolean network was applied to the three distinct characteristics microarray time-series data, whose resulting networks were compared at a setting scenario: mean discretisation method and stringency at 100 percent. The studied datasets included (1) Galactose system (data from [13]), (2) circadian clock system under constant light (data from [14]), and (3) circadian clock system under light/dark cycle (data from [15]).

Besides the aforementioned factors, the performance of the Boolean network inference is believed to be very much dependent on the quality and resolution of data. We, therefore, explored further on how such varying characteristics of data affect the inferred network. Since we here focused on the time-series data of the oscillatory gene expression, the *quality* and *resolution* of data were defined by the amplitude of the expression profile and the number of datapoints, respectively. The amplitude in this study was estimated by the average distance between the peak and trough of the oscillation profiles (Equation 10). For the sake of a variety in characteristics of the data employed in the study, we can explicitly plot the performance of the inferred networks against the number of datapoints (Figure 6A) and the amplitude of the time-series (Figure 6B). Note that the network performance presented in Figure 6 belongs to the results of inference using the constraint-based Boolean regime at the highest level of stringency (100 percent). Figure 6A shows that more number of datapoints can improve the *accuracy*, *precision* and *specificity* of the inferred network, but may limit its *sensitivity*. On the contrary, the highest amplitude data shows the lowest *accuracy*, *precision* and *specificity*, while possesses the highest network *sensitivity* (Figure 6B). At a first glance, it appears that the effect of the amplitude on the network performance is opposite to that of the amount of the datapoints, yet it might be an artifact. The apparently poor network performance of the highest amplitude data (Clock-Blasing) can be a consequence of very small amount of datapoints rather than the effect of the amplitude. The result of the combinatorial effects of such factors observed in these datasets may suggest that the number of datapoints is relatively more influence on the performance of the Boolean network inference than the amplitude of the data. Specifically, *accuracy*, *precision*, and *specificity* of the inferred network rely enormously on the number of datapoints, while the *sensitivity* is likely more dependent on the amplitude of the time–series.

#### The inferred Boolean network of the galactose system

**Figure 7:**
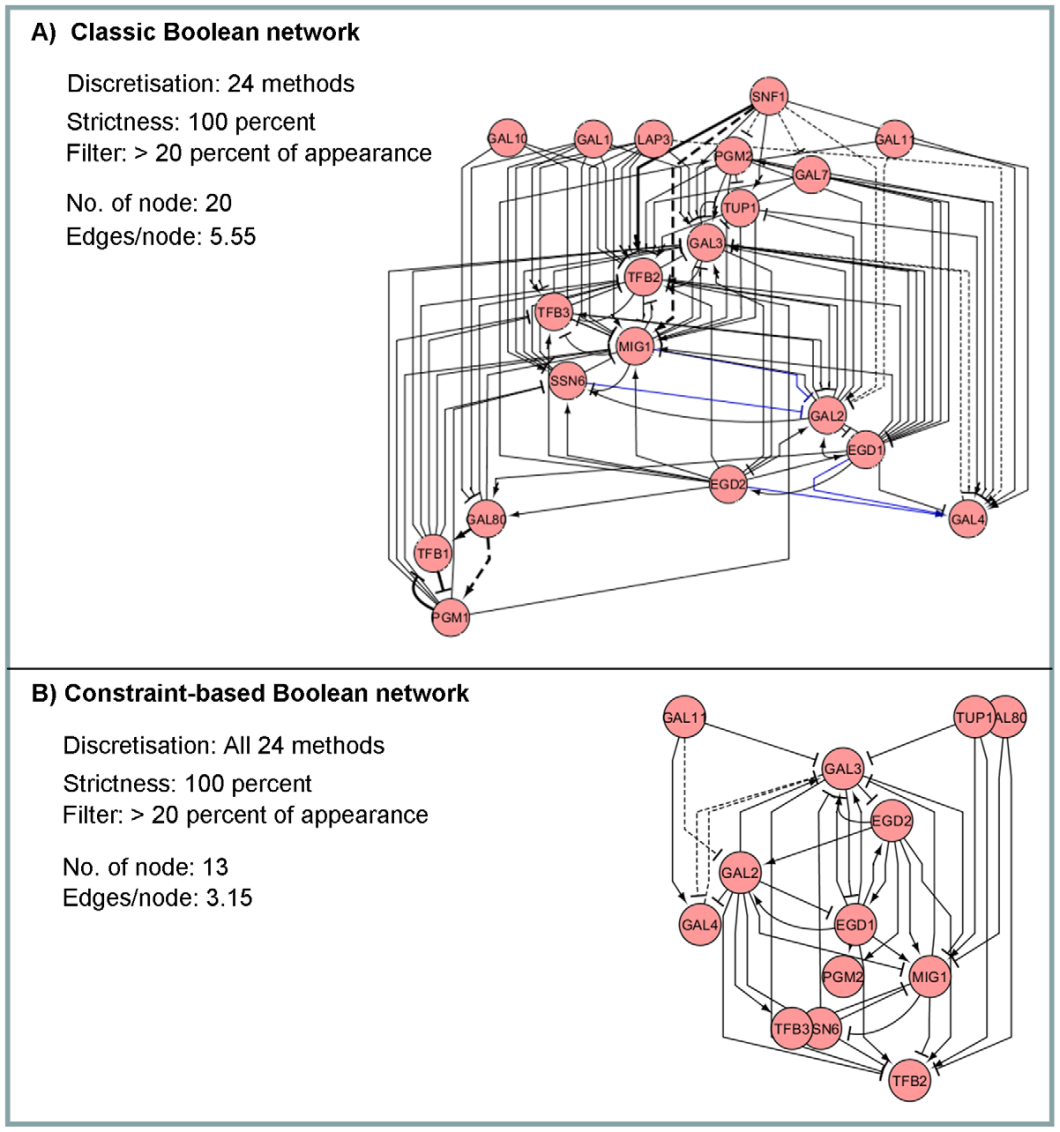
Classical and constraint-based Boolean networks of the galactose system. The figure depicts the galactose networks inferred by (A) the classical and (B) constraint-based Boolean network. The *master Boolean networks* (see Methods) presented were the results of combining 24 individual networks of using 24 discretisation varieties at the highest level of stringency (100 percent).

The example of the inferred genetic networks is illustrated in Figure 7 whereby the networks of the galactose system inferred by the classical Boolean (Figure 7A) were compared with the one inferred by the constraint-based Boolean (Figure 7B). The example networks presented are a *master Boolean network* (see Methods) describing regulation in the galactose system derived from a summation of the 24 Boolean networks of using 24 different discretisation varieties employed in Figure 2. A gene pair that is present in at least four individual networks (4/24 or ∼20%) was kept in the final scheme. With the same setting for the other Boolean parameters, the two inferred networks of the galactose system obviously show different complexity. Incorporating the constraints into the Boolean algorithm significantly reduces the complexity of the inferred network (*i.e.* from 20 nodes and 5.55 edges/node to 13 nodes and 3.15 edges/node), leading to a more understandable result. It might be claimed that our protocol intended to provide a more comprehensible network instead of aiming at the rather complete network which is usually highly complicated due to an attempt to include immense data into the network. The network scheme also demonstrated that the constraints enable the elimination of false positive, providing the less complicated network with more accuracy. By classifying the false positive prediction into two classes, which are prediction of unidentified data and wrong prediction, we showed in our case study that the majority of the apparent false positive is Boolean prediction of unknown or unidentified event instead of a certainly wrong prediction (Figure S7). It is noteworthy that the predicted regulation that falls into this class of false positive may be a result of lacking of the background knowledge to develop the relatively more complete reference network. In Figure 7, such two classes of false positive are differently denoted as black solid lines and black dash lines, respectively. Despite the explicit advantage of the constraints, it may cause a loss of true positive as seen in the example study (diminution of blue solid lines in Figure 7B). One explanation could be because of the elimination of the complex regulations, *e.g.* self-regulation, indirect regulation, and feedback control, resulting from a highly restricted constraint. Therefore, the use of the constraints to improve the efficacy of Boolean algorithm needs to be done with care.

### 2. A practical guideline for efficient use of Boolean-based method

The following suggestions may be considered as a guideline for Boolean analysis regime for network inference application:

- Mean, mid-range, and *T_o_*_mean±*x*SD (at *x* = 0) are comprised of a primary group of discretisation methods which are recommended to be the first choice of data transformation method. This suggestion was concluded based on the advantages in their compatibility to various types of data and their ability to provide a good inferred network.
- Constraints (derived from general facts and prior biological knowledge) should be commonly incorporated into the Boolean analysis regime to increase the *accuracy*, *precision* and *specificity* of inferring networks, yet the overflow of which may cause a reduction in network *sensitivity*.
- The highest level of stringency (100 percent) for Boolean function assignment can be naively used as a default setting for Boolean analysis regime. This suggestion is valid for a time-series containing up to twelve datapoints. For higher resolution data, the majority rule that is assigning the Boolean function in accordance with the majority population can be employed to relax the level of stringency as often found in literature of Boolean network inference.

Furthermore, we presented a rational way to resolve the multi-solution problem of the Boolean network inference resulting from a choice of discretisation methods. Although we identified a primary choice of methods that could alleviate the problem, the unique solution for a studied dataset cannot be concluded. Since the best discretisation method for a dataset is unfeasibly defined and neither of which is good for all datasets, we strategically inferred a single solution network of the system through the integration of all possible solutions. The individual networks resulting from different discretisation methods were merged into one genetic map so called *master Boolean network*, in which the frequency of appearance of the interaction, denoted as the thickness of the edge in the hypergraph, reflects the confidence of such interaction in the inferred network. This approach instead took the advantage of having data from multiple discretisation methods to increase the reliability of the inferred network.

In conclusion, our study provided a guideline that facilitates the use of Boolean algorithm in the network inference application as well as the approach to acquire the master network for the investigated system or dataset.

### 3. Making use of the guideline to infer the genetic networks of the circadian clock system

To further explore the advantage of the established guideline, more examples of its usage will be described. In this section, we looked closely to the resulting networks of the circadian clock system derived from Boolean network inference protocol on the basis of the following settings:

- Constraints (Figure S8):
*Conceptual constraints* are the list of enzymatic genes (*i.e. PGI1*, *TPI*, *PGK*, and *PGM*) that hypothetically have no feedback regulation to the regulatory genes.
*Specific constraints* are the non-existent relationships implied by the literature, including the feedback regulation from flowering pathway (*CO*, *FT*, and *SOC1*) to the circadian clock (*CCA1*, *ELF4*, *GI*, *LHY*, *PRR5*, *PRR7*, and *TOC1*).
- Level of stringency for Boolean function assignment: highest or 100 percent.
- Discretisation methods: 23 discretisation varieties presented in Figure 2 (excluding sign of log_2_ratio).
- Filter: *consistency* >20 percent (5/23), *i.e.* the *master Boolean networks* were drawn from the relationships of gene pairs that are highly consistent (>20 percent) among the results of using different discretisation methods.

**Figure 8:**
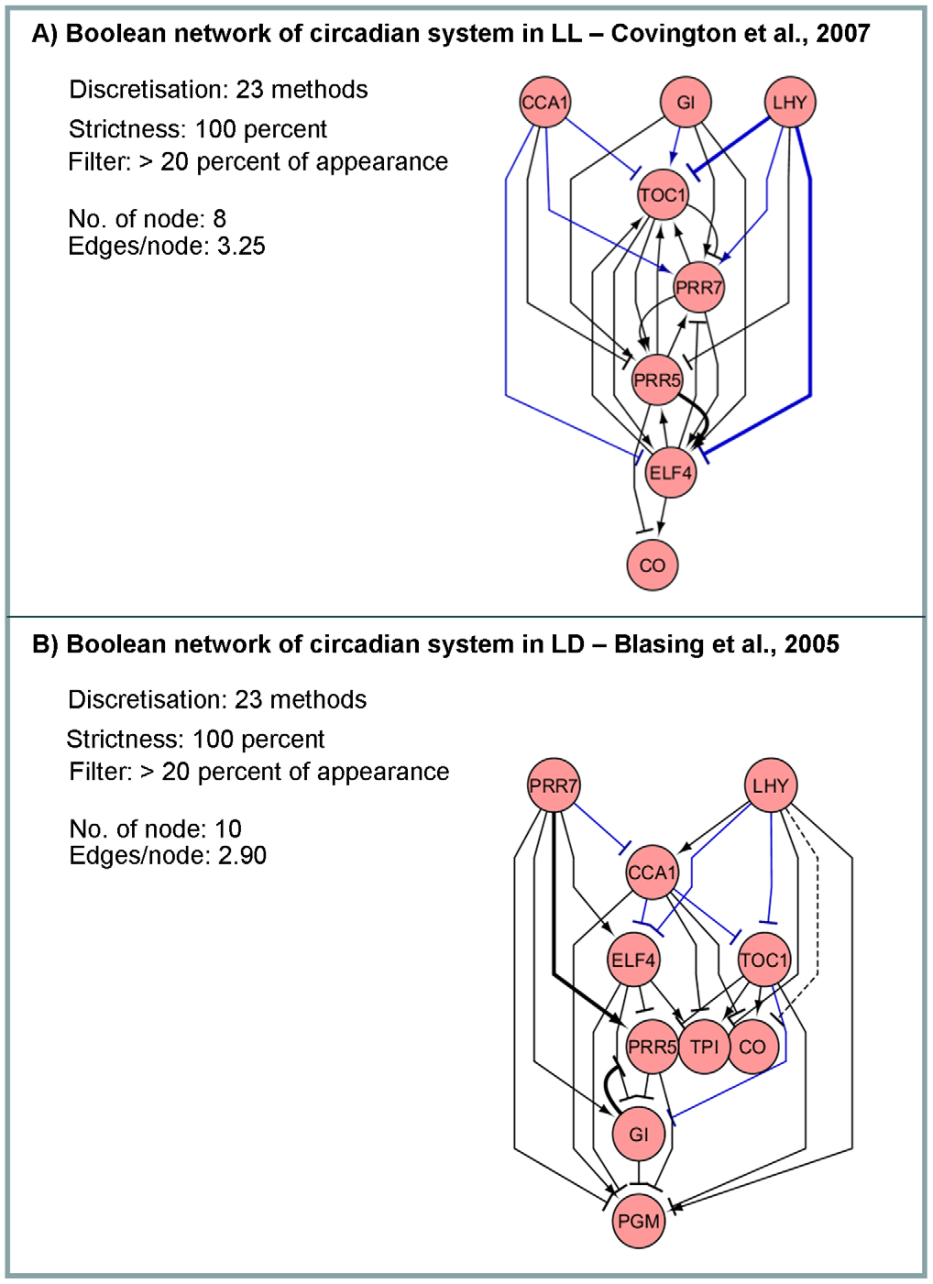
Boolean networks of circadian clock systems. Microarray data measured under different light conditions were analysed by the constraint-based Boolean approach resulting in the inferred networks of circadian clock system (A) under constant light (LL; [14]) and (B) under constant light (LD; [15]). The *master Boolean networks* (see Methods) presented were the results of combining 23 individual networks of using 23 discretisation varieties at the highest level of stringency (100 percent).

Figure 8 depicts the *master Boolean networks* of the circadian clock that represent the regulation of the systems under continuous light (LL; Figure 8A) and light/dark cycle (LD; Figure 8B) conditions in accordance with the employed microarray data [14], [15]. Like the galactose system, the inferred networks are relatively simpler and easier to understand than those derived from the classical Boolean algorithm (unpublished data). An alternative setting was also performed to ensure the goodness of our choice of selection. By reducing the level of stringency to 80 percent, while maintaining the other settings, the resulting network has significantly high false positive and becomes more complicated (*e.g.* for the LL condition: from 8 nodes and 3.25 edges/node to 11 nodes and 7 edges/node; and for the LD condition: from 10 nodes and 2.90 edges/node to 12 nodes and 4.92 edges/node; Figure S9). Therefore, we hereafter used the inferred network of circadian clock system in Figure 8 for discussion. Each network was firstly discussed with an advice from literature. Then, the two networks were compared in both theoretical and biological aspects.

Circadian clock is known as a gene network generating about 24-h rhythm. It is found in diverse organisms, including *Arabidopsis* model plant. Though the components comprised of the network have been studied continuously and they are not fully known yet, there is at least a group of genes that have been claimed to be involved in the rhythm generation: *CIRCADIAN CLOCK ASSOCIATED 1 (CCA1*; [30]), *LATE ELONGATED HYPOCOTYL (LHY*; [31]), *TIMING OF CAB EXPRESSION 1 (TOC1*; [25]), *PSEUDO-RESPONSE REGULATORS (PRRs*; [32], [33]), *GIGANTEA (GI*; [30], [34], [35]), *EARLY FLOWERING 4 (ELF4*; [26]). Regarding the decades of study, the roles and positions of these genes within the circadian clock network have been reviewed by [25] and [37].

By comparing the inferred networks (Figure 8) to the reference scheme redrawn from [19] (Figure S1B2), we found certain matches, which were called true positive prediction, marked as blue solid lines in Figure 8. Components of negative feedback regulation in the circadian clock system were correctly predicted, yet the complete loop was captured in neither networks. For example, both networks successfully identify a component of the well-known morning loop of *Arabidopsis* circadian clock [25], [26], the inhibition of *CCA1* and *LHY* on *TOC1* and *ELF4*. For the evening loop of *TOC1* and *GI* initiated by [34], the inhibition of *TOC1* on *GI* was only found in the analysis of Covinton *et al*'s data, while the reciprocal activation was presented just in the network of Blasing *et al*. Apart from the correct prediction of known regulation, we also paid our attention to the inference of previously unidentified gene relationships. The positive prediction in this group was classified as false positive relying on the current data, but these relationships in fact might exist in the real situation. The instances of the interesting predictions are the activation of *PRR7* on *PRR5*, the activation of *TOC1* on *CO*, and the activation of *PRR7* on *ELF4*. The relationship of *PRR7* and *PRR5* has been mentioned in various works [26], [33] as well as in the recent model of *Arabidopsis* circadian clock where *PRR5* as a candidate of *NI* was proposed to be activated by *PRR7* [36]. Similarly, the activation of *TOC1* on *CO* has been suggested in both experimental and modelling scenarios [37], [38], despite no direct validation. In contrast, very few biological evidence for the relationship between *PRR7* and *ELF4* is available.

Since the employed datasets are measures of gene expression profiles of *Arabidopsis* under different light conditions (*i.e.* LL and LD), the distinction in the resulting networks may infer the condition-specific regulation. Such a comparative approach is generally used for insight into the results, but it is effective only if the results are derived from good controlled data. Thus, we coped with the divergence of our data characteristics by considering only the highly consistent relationships (marked as a thick line in Figure 8). The following cases are examples of information extracted from the comparison between the inferred networks. *GI* positively regulates *PRR5* under the LL condition, while negatively regulates *PRR5* under the LD condition. The genetic linkage between *PRR5* and *GI* was implied in the study of Kawamura [39], where *gi prr5* double mutant was developed. They proposed various scenarios of relationships of those genes, including linear and parallel regulations; however, the light-dependent regulation was not clearly reported [39]. It has been reported that *PRR5* and *GI* may relate via an F-Box protein ZTL, whereby the inhibition of *GI* on *PRR5* may be described by GI stabilising ZTL protein [40] which in turn targets PRR5 for degradation [41]. Another observation is the positive feedback loop of *PRR5* and *ELF4* under the LL condition which apparently becomes a single inhibition relationship under the LD condition. The similar result was shown in the network inferred by the intensive Bayesian algorithm [7]. This suggests that the *PRR5* and *ELF4* are one of the most closely related genes among the studied gene set, yet the direction of the regulation is still ambiguous.

## Discussion

This work was motivated by a question of how to improve the Boolean-based approach effectively. We focused our interest to a practical approach to increase the *accuracy*, *precision*, *specificity* and *sensitivity* with minimum requirements of computational expenditure and sophisticated codings. The logical first step to answer the question is to explore the factors affecting Boolean network performance. Here, we studied the effect of three basic factors that have often been set intuitively in the Boolean-based analysis: discretisation methods, constraints, and stringency of Boolean function assignment. Our results suggest that constraints have pivotal effect on the Boolean network performance over the other two factors (Figures 3 and 4). Incorporating the biological constraints into the Boolean-based regime does not only improve the network performance through the reduction of false positive, but it also lessens the variation of the results due to the choice of discretisation methods and level of stringency. As the constraints are mostly derived from prior knowledge, and more constraints applied to the Boolean-based regime result in more accurate resulting network [12], it suggests that the background knowledge or information is necessary to increase the correctness of the Boolean network inference. It might be arguable that integrating prior knowledge into the inference framework is not an absolute bias; rather it provides fact-based criteria to eliminate the excessive false positive from the innocent Boolean analysis. In other words, we claimed that Boolean-based method is not very useful for analysis the completely unknown system, yet it is doable in theory.

An experience in the analysis allowed us to establish the general guideline for Boolean-based method employment in the application of network inference. The guideline provides decision support information for setting up the protocol of Boolean analysis, including a guide for selecting a proper discretisation method, a guide for implementing biological constraints, and a guide for setting the stringency of the Boolean function assignment (see Section 2 in Results). The advantage of the guideline is not only a cue for setting a protocol, but it helps to reduce the points to be concerned under the environment of Boolean-based analysis. For instance, we can pay less attention to the setting of stringency of Boolean function assignment if the constraint-based Boolean regime is employed (Figure 4). For a matter of generality, this guideline was formulated from the results of the study based upon three datasets having distinct characteristics: galactose system from [13] – seven datapoints and 1.25 in amplitude; Circadian clock in LL condition from [14] – twelve datapoints and 1.39 in amplitude; Circadian clock in LD condition from [15] – six datapoints and 3.11 in amplitude; Figures 5 and 6). It is hence expected to be applicable to various characteristics of data, yet not all cases. An exception of the guideline can be postulated as a system that is beyond our study, such as time-series data with very low resolution.

Besides the guideline, we proposed the strategy to define a unique solution network, called *master Boolean network*, from the Boolean-based analysis regime. The idea is to integrate all resulting networks derived from different discretisation methods or settings into a single map, whereby the consistency of the relationships between gene pairs among the integrated results is highlighted by the thickness of the edge and the low consistent relationships can be filtered out. Through this approach, we can indicate the confidence of the inferred gene relationships relying on the consistency. It is the additional information apart from the previous Boolean analysis. The *master Boolean network* is thus an informative presentation of a resulting network containing the inferred gene relationships with a level of confidence (*e.g.* Figures 7 and 8).

According to the guideline and the concept of master network, we illustrated their usefulness in the study of *Arabidopsis* circadian clock (Section 3 in Results). The two sets of microarray time-series data that were measured under different light exposure were employed in the study, by aiming at the genetic network of the *Arabidopsis* circadian clock. The results, in Figure 8, show that performing Boolean-based analysis in accordance with the guideline successfully inferred the network of the *Arabidopsis* circadian clock with a certain degree of true positives and few false positives (wrong prediction type) with respect to the reference network in Figure S1B2 [19]. We observed that nearly all correct predictions are involved in the regulation of *CCA1* and *LHY* genes. It is possibly because of a clear expression profile of such genes incorporated with their fruitful information. Unlike *LHY* and *CCA1*, the relationship related to *TOC1* was not well predicted, though it is one of the most studied genes in the circadian clock system. This occurrence is not totally unexpected, since *TOC1* expression profile is usually low in amplitude, especially under LL condition [42].

Through the comparison between the networks (Figures 8A and 8B), the consistent gene relationships may reflect the principle circuit of the circadian clock system, while the differences may indicate the regulation underlying a particular light condition. The given confidence of the results is exploited here to define our scope of interest. By looking at the high confidence results, we pointed out the distinct relationships of the two gene pairs inferred under LL and LD conditions: *PRR5* – *GI* and *PRR5* - *ELF4*. Other independent studies supporting these predicted relationships were also discussed here. As *PRR5*, *GI* and *ELF4* are among the most interesting genes in the current circadian clock research; our findings may be used as a seed for hypothesis development and experimental design to get more insight into the function of those genes in the clock network.

In brief, Boolean-based method, known as a simple approach for inferring a network from the expression data with the least requirement, was demonstrated here that the efficiency of the method in terms of prediction power relies very much on the background information. Without prior knowledge, the results may inevitably include a number of false positives which become a major weakness of the method. We, thus, recommend that the good way to improve Boolean-based method is to integrate the prior knowledge into its framework. In particular, this method may help increase the prediction power of Boolean network inference significantly in a large-scale analysis which is a real advantage of Boolean-based method.

## Supporting Information

Figure S1 **Expression profiles of genes of interest in the studied datasets and the reference networks of the systems under study.** The expression profiles were obtained from the three independent microarray experiments: (A1) log_2_ ratio of galactose-involved genes in *S. cerevisiae* [13], (A2) (left) log_2_ ratio and (right) intensity of circadian-clock-involved genes in *Arabidopsis* [14], and (A3) (left) log_2_ ratio and (right) intensity of circadian-clock-involved genes in *Arabidopsis* [15]. According to the existing data, the simplified genetic network of the (B1) galactose system and (B2) circadian system were drawn as the reference networks in Boolean network analysis.
(TIF)

Figure S2 **The effect of constraints on the Boolean network inference regime.** The performances of constraint-based Boolean network for circadian system (data from [14]) were compared with the classical Boolean network inference: (A) – accuracy; (B) – precision; (C) sensitivity; (D) specificity; black – non-constrained and hatch – constrained).
(TIF)

Figure S3 **The effect of constraints in the Boolean network inference regime.** The performances of constraint-based Boolean network for circadian system (data from [15]) were compared with the classical Boolean network inference: (A) – accuracy; (B) – precision; (C) sensitivity; (D) specificity; black – non-constrained and hatch – constrained).
(TIF)

Figure S4 **The effect of constraints on the Boolean network prediction.** The Boolean networks of Galactose system (data from [13]) were computed using classic and constraint-based Boolean network inference algorithms. The prediction results from both methods were compared: (A) TP - true positive, (B) TN - true negative, (C) FP – false positive, (D) FN, - false negative; black – non-constrained and hatch – constrained).
(TIF)

Figure S5 **The effect of level of stringency of Boolean function assignment.** The performances of constraint-based Boolean network for circadian clock system (data from [14]) (right; A,C,E,G) were compared with those of the classical Boolean network (left; B,D,F,H) inference under different degrees of stringency of Boolean function assignment: black – 100, hatch – 80, and grey – 50 percent).
(TIF)

Figure S6 **The effect of level of stringency of Boolean function assignment.** The performances of constraint-based Boolean network for circadian clock system (data from [15]) (right; A,C,E,G) were compared with those of the classical Boolean network (left; B,D,F,H) inference under different degrees of stringency of Boolean function assignment: black – 100, hatch – 80, and grey – 50 percent).
(TIF)

Figure S7 **Classification of false positive.** False positives appearing in the galactose systems were divided into two classes: white – prediction of the unidentified event and black – wrong prediction; where (A) determined by non-constraint and (B) determined by constraint based methods.
(TIF)

Figure S8 **The input file of prior knowledge for constraints-based Boolean network of circadian clock system.** The constraints incorporated into the Boolean network can be classified into two types: (A) Conceptual constraints (B) Specific constraints.
(TIF)

Figure S9 **Boolean networks of circadian clock systems.** Microarray data measured under different light conditions were analysed by the constraint-based Boolean approach resulting in the inferred networks of circadian clock system (A) under constant light (LL; [14]) and (B) under constant light (LD; [15]). The presented networks were the results of combining 23 individual networks of 23 discretisation methods derived by using level of stringency at 80 percent.
(TIF)

Table S1 **Similarity scores of the profiles discretised from various methods.**
(TIF)
