# Supplementary figures and images for: Analysis and Practical Guideline of Constraint-Based Boolean Method in Genetic Network Inference
Source: PLoS One. 2012 Jan 17;7(1):e30232. doi: 10.1371/journal.pone.0030232 (PMC3260258; doi:10.1371/journal.pone.0030232)

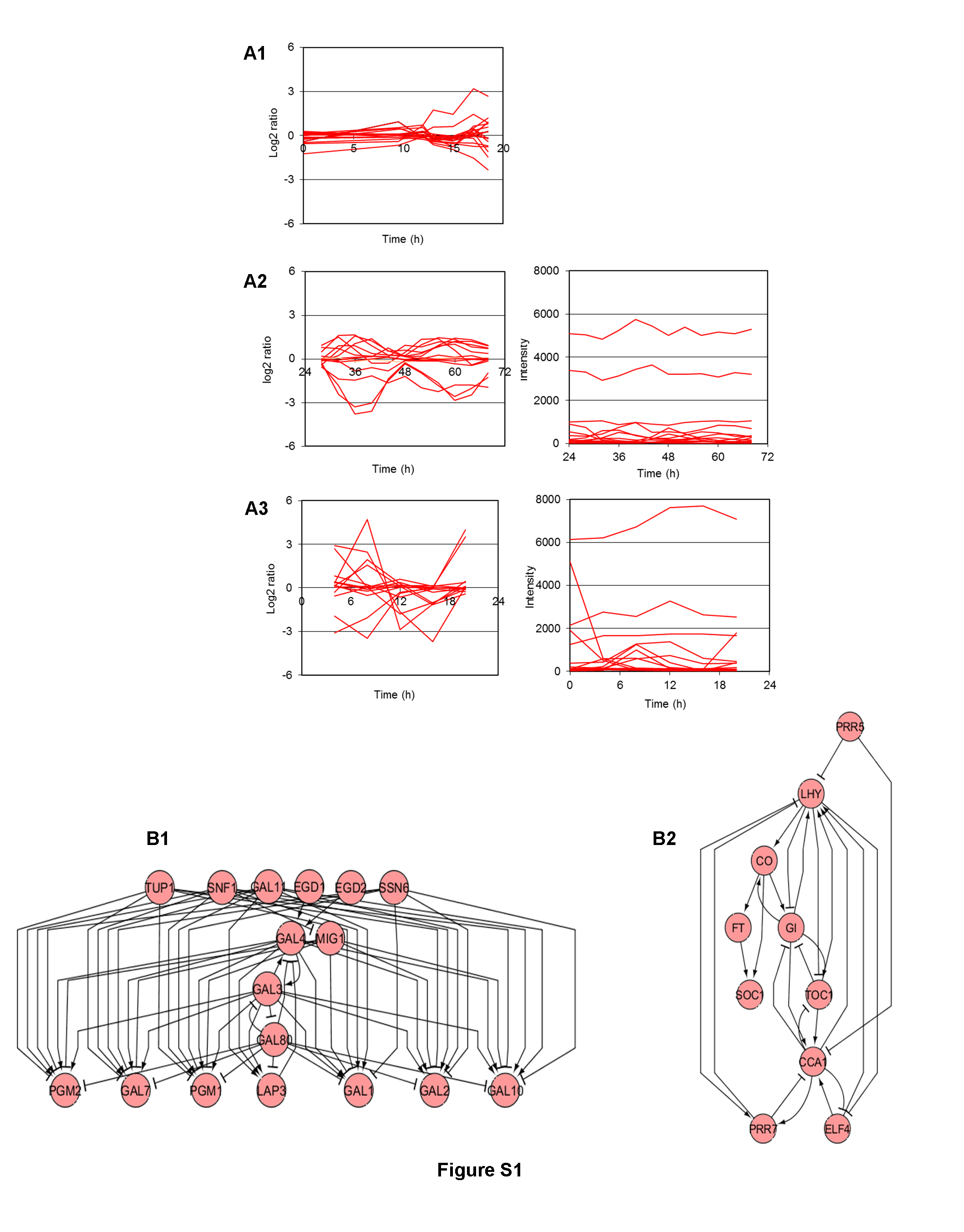

Supplement: Figure S1 — Expression profiles of genes of interest in the studied datasets and the reference networks of the systems under study. The expression profiles were obtained from the three independent microarray experiments: (A1) log2 ratio of galactose-involved genes in S. cerevisiae [13], (A2) (left) log2 ratio and (right) intensity of circadian-clock-involved genes in Arabidopsis [14], and (A3) (left) log2 ratio and (right) intensity of circadian-clock-involved genes in Arabidopsis [15]. According to the existing data, the simplified genetic network of the (B1) galactose system and (B2) circadian system were drawn as the reference networks in Boolean network analysis. (TIF) [file pone.0030232.s001.tif]

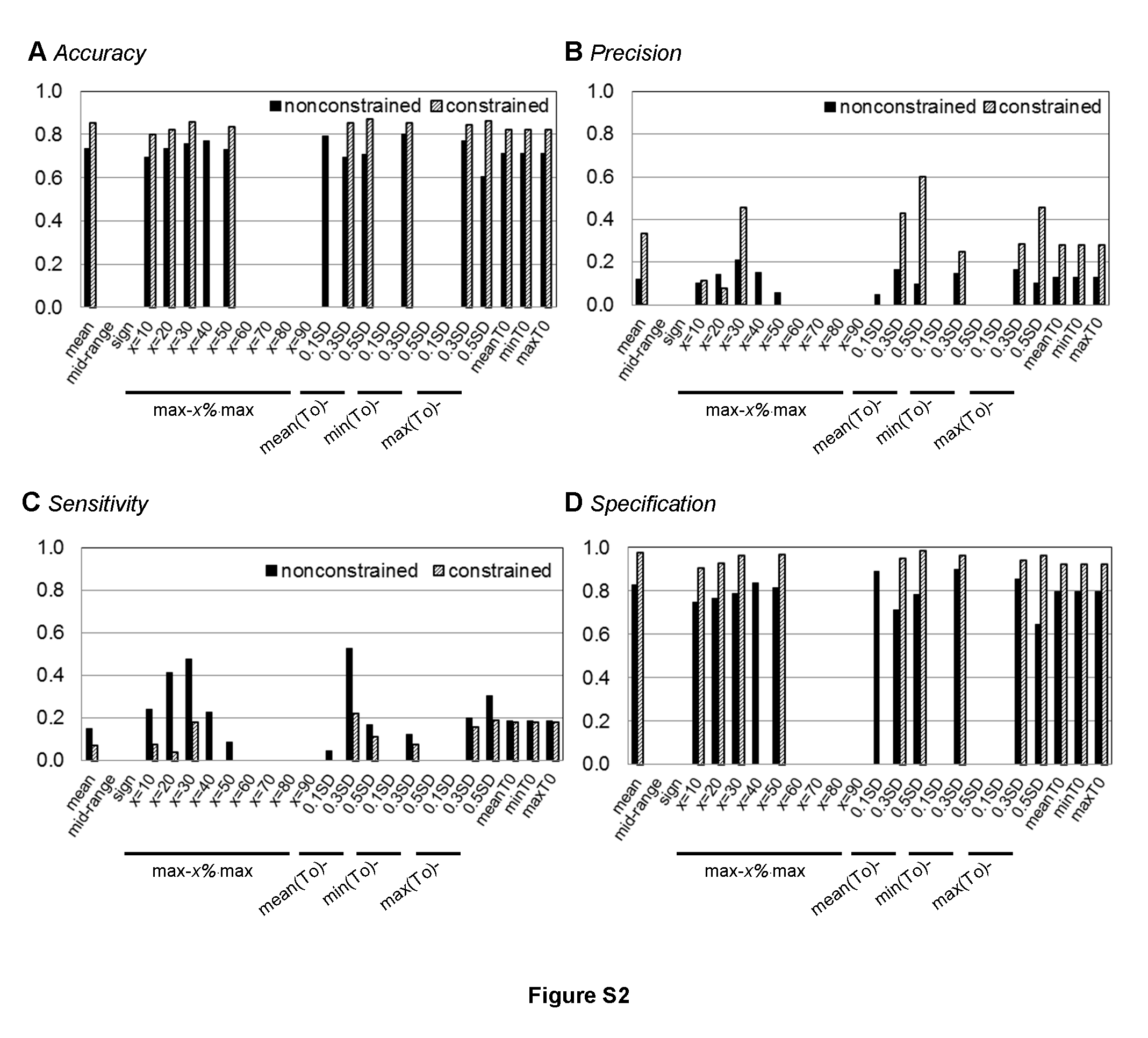

Supplement: Figure S2 — The effect of constraints on the Boolean network inference regime. The performances of constraint-based Boolean network for circadian system (data from [14]) were compared with the classical Boolean network inference: (A) – accuracy; (B) – precision; (C) sensitivity; (D) specificity; black – non-constrained and hatch – constrained). (TIF) [file pone.0030232.s002.tif]

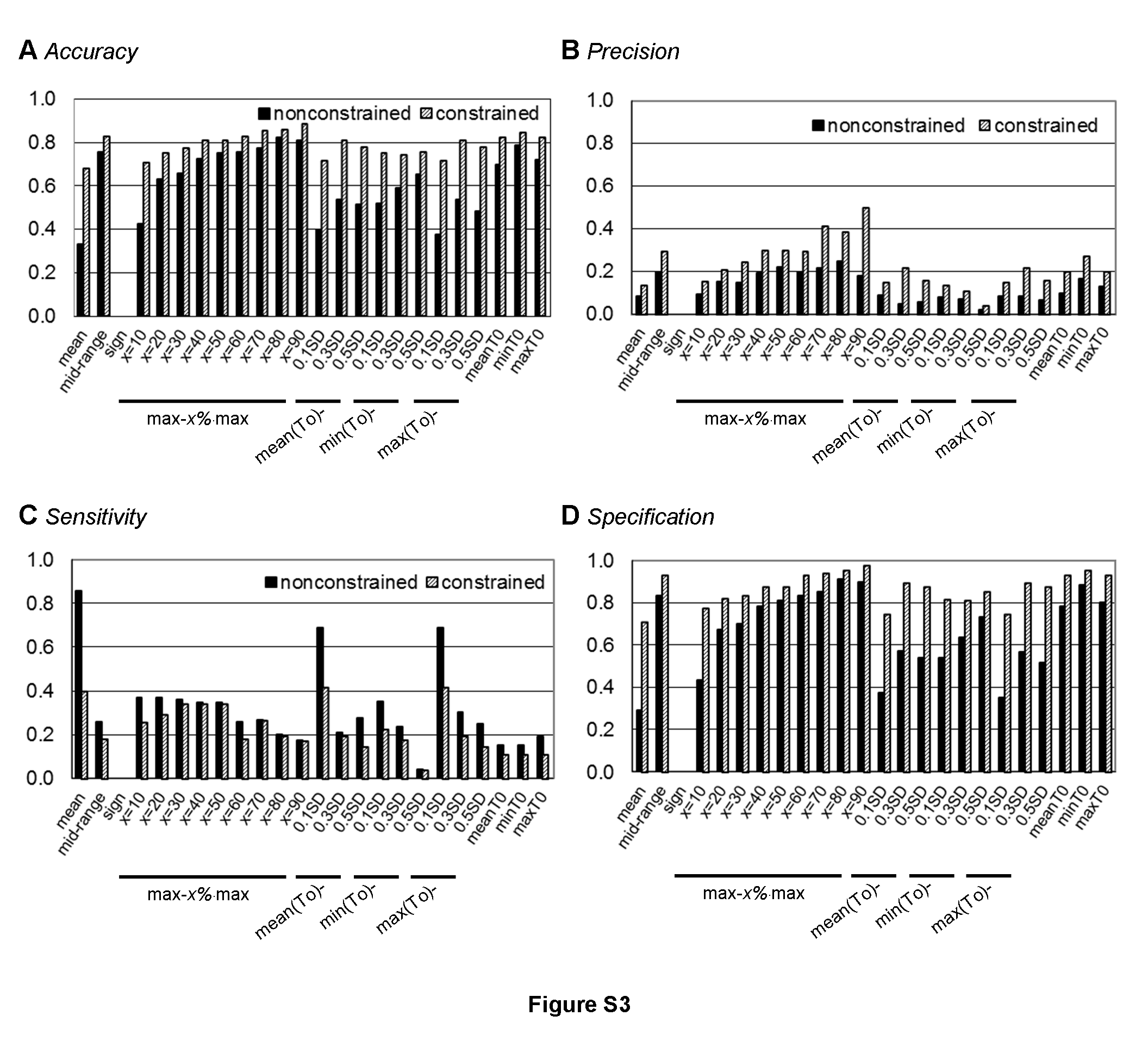

Supplement: Figure S3 — The effect of constraints in the Boolean network inference regime. The performances of constraint-based Boolean network for circadian system (data from [15]) were compared with the classical Boolean network inference: (A) – accuracy; (B) – precision; (C) sensitivity; (D) specificity; black – non-constrained and hatch – constrained). (TIF) [file pone.0030232.s003.tif]

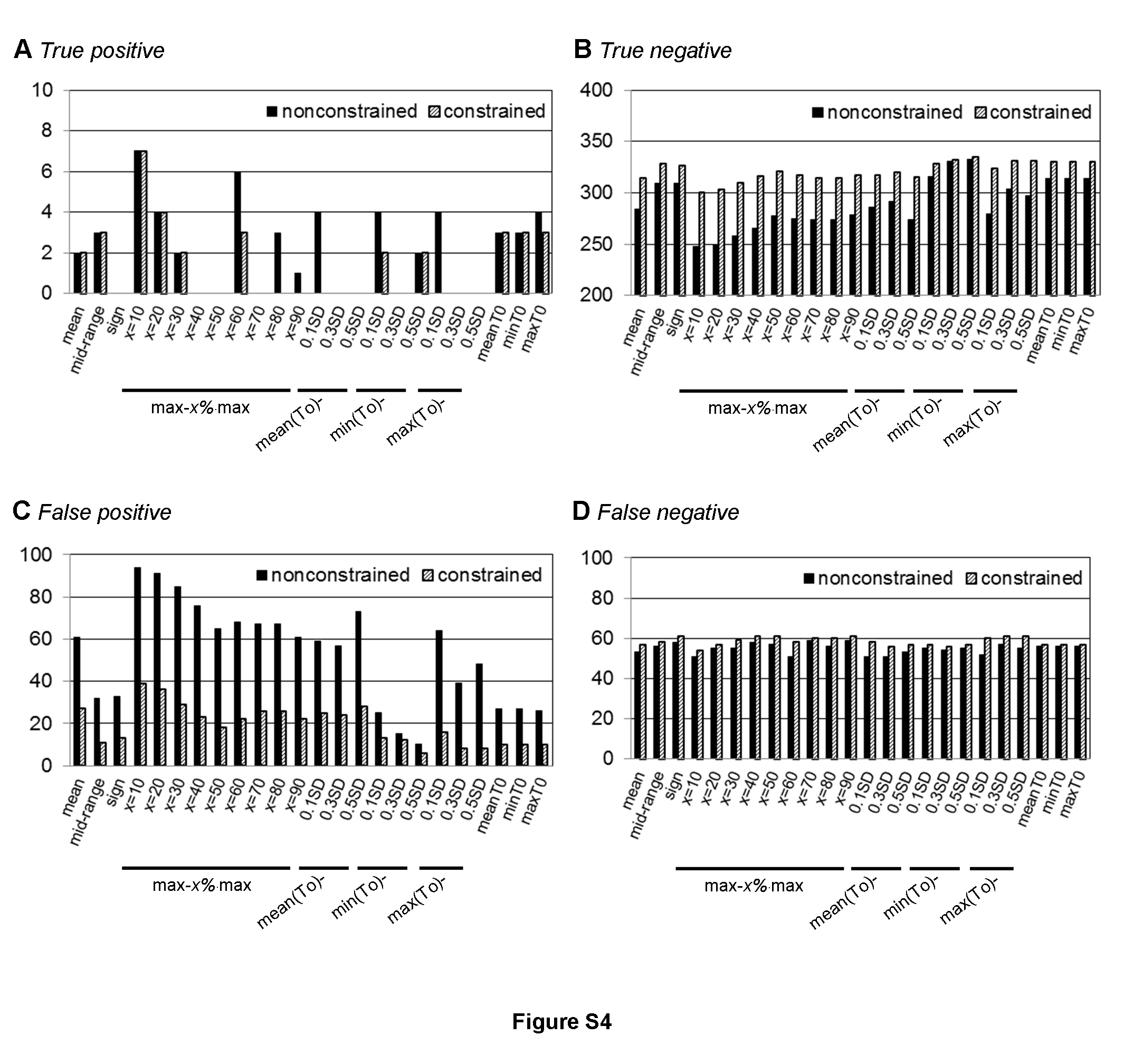

Supplement: Figure S4 — The effect of constraints on the Boolean network prediction. The Boolean networks of Galactose system (data from [13]) were computed using classic and constraint-based Boolean network inference algorithms. The prediction results from both methods were compared: (A) TP - true positive, (B) TN - true negative, (C) FP – false positive, (D) FN, - false negative; black – non-constrained and hatch – constrained). (TIF) [file pone.0030232.s004.tif]

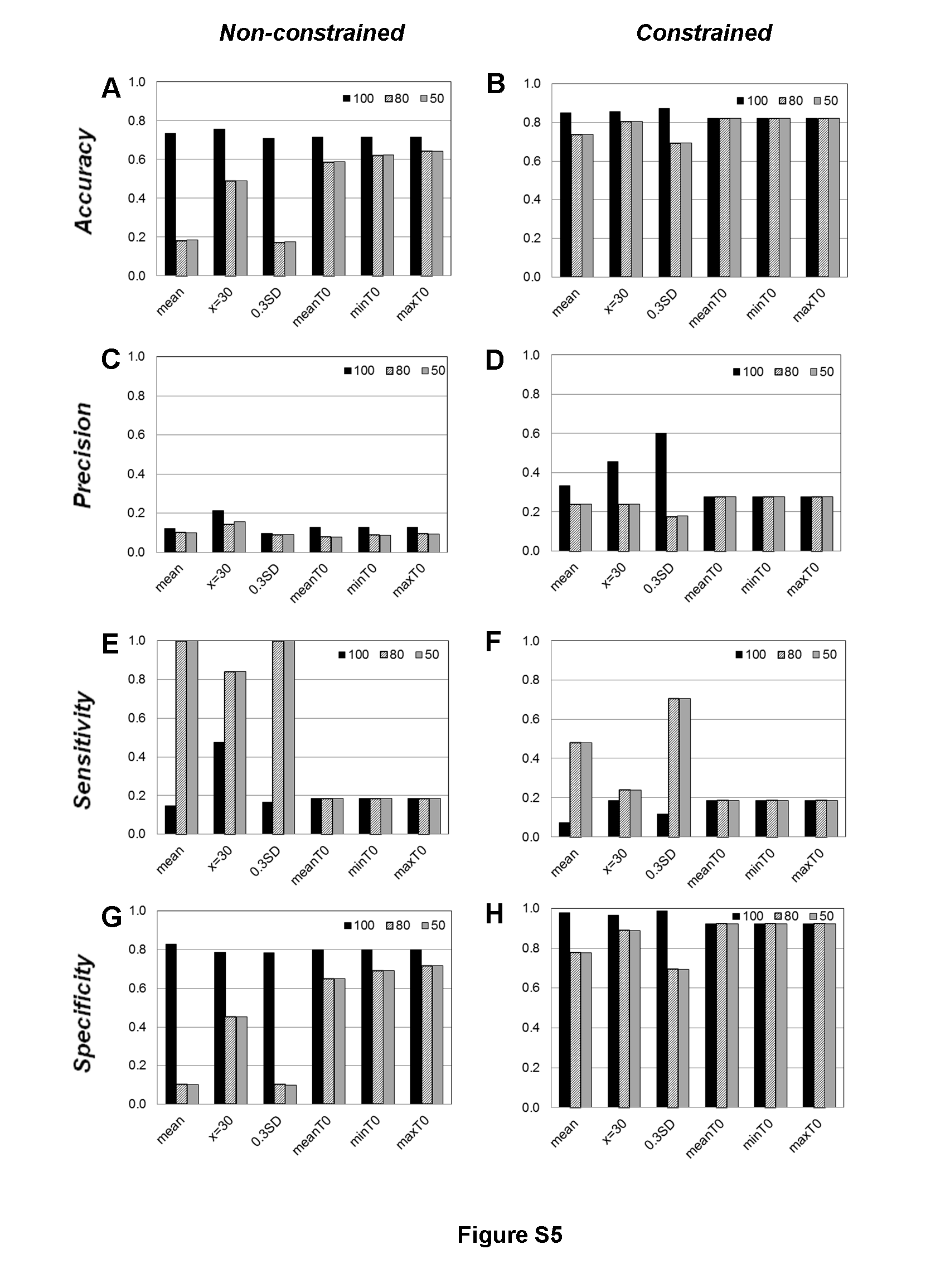

Supplement: Figure S5 — The effect of level of stringency of Boolean function assignment. The performances of constraint-based Boolean network for circadian clock system (data from [14]) (right; A,C,E,G) were compared with those of the classical Boolean network (left; B,D,F,H) inference under different degrees of stringency of Boolean function assignment: black – 100, hatch – 80, and grey – 50 percent). (TIF) [file pone.0030232.s005.tif]

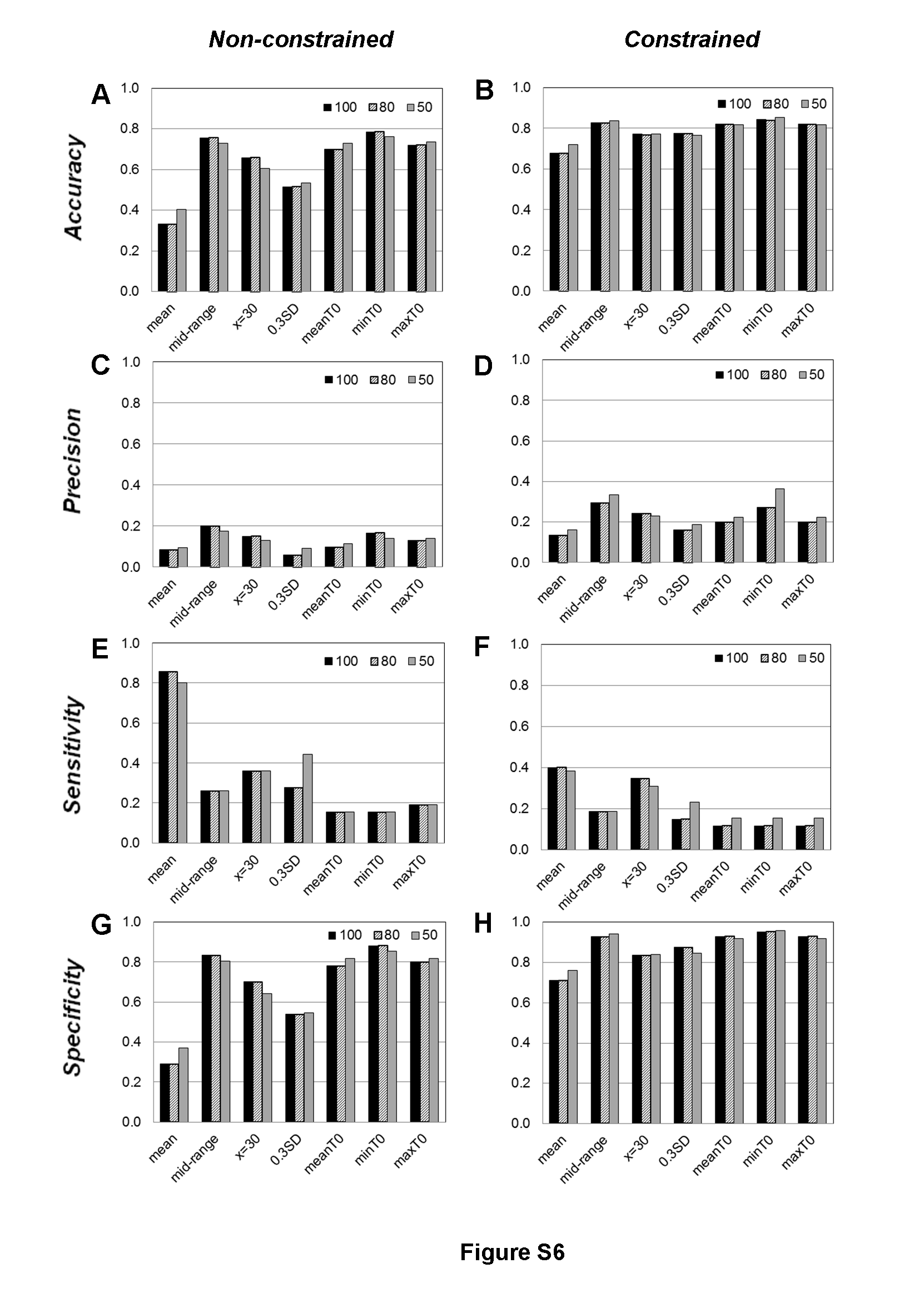

Supplement: Figure S6 — The effect of level of stringency of Boolean function assignment. The performances of constraint-based Boolean network for circadian clock system (data from [15]) (right; A,C,E,G) were compared with those of the classical Boolean network (left; B,D,F,H) inference under different degrees of stringency of Boolean function assignment: black – 100, hatch – 80, and grey – 50 percent). (TIF) [file pone.0030232.s006.tif]

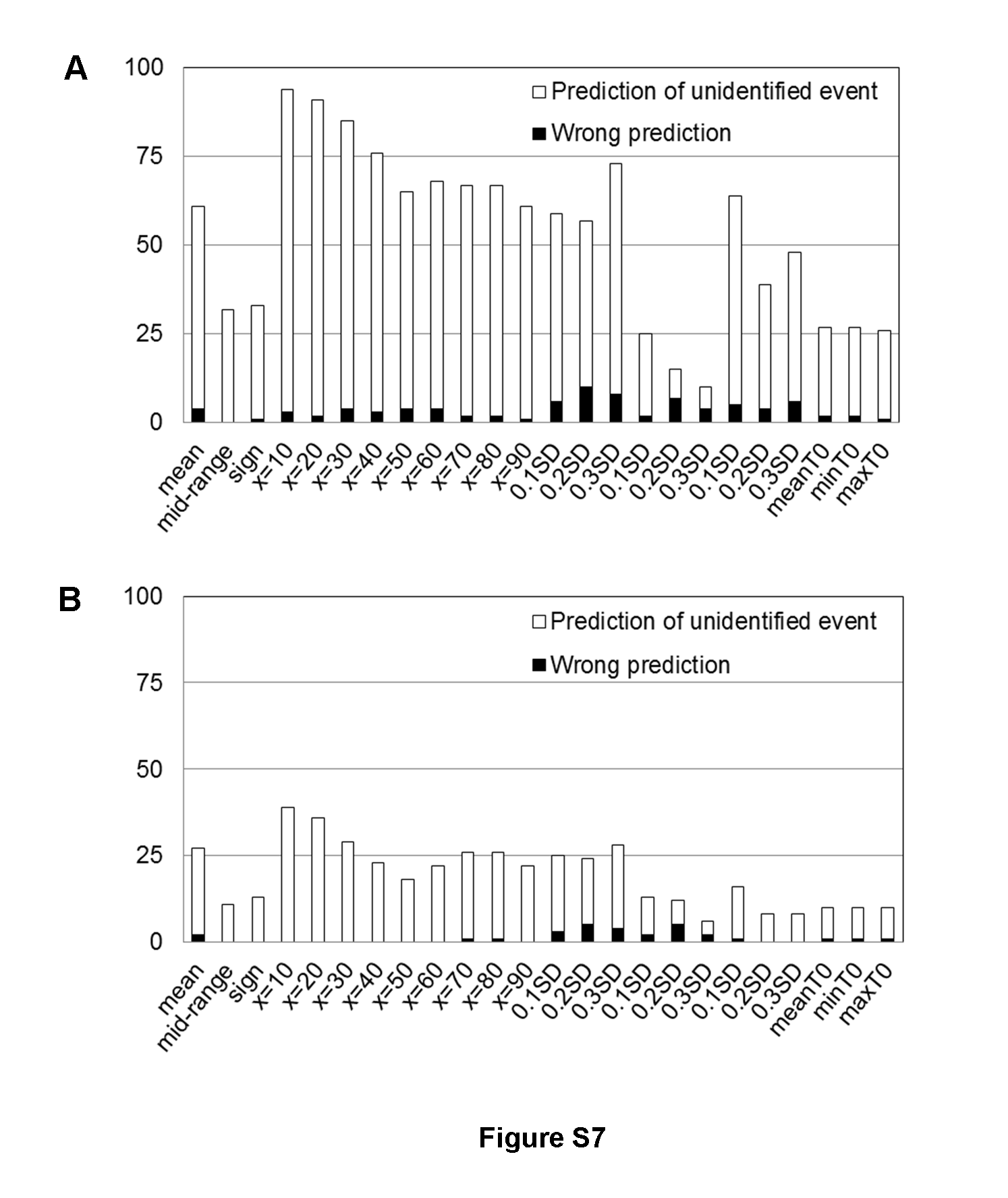

Supplement: Figure S7 — Classification of false positive. False positives appearing in the galactose systems were divided into two classes: white – prediction of the unidentified event and black – wrong prediction; where (A) determined by non-constraint and (B) determined by constraint based methods. (TIF) [file pone.0030232.s007.tif]

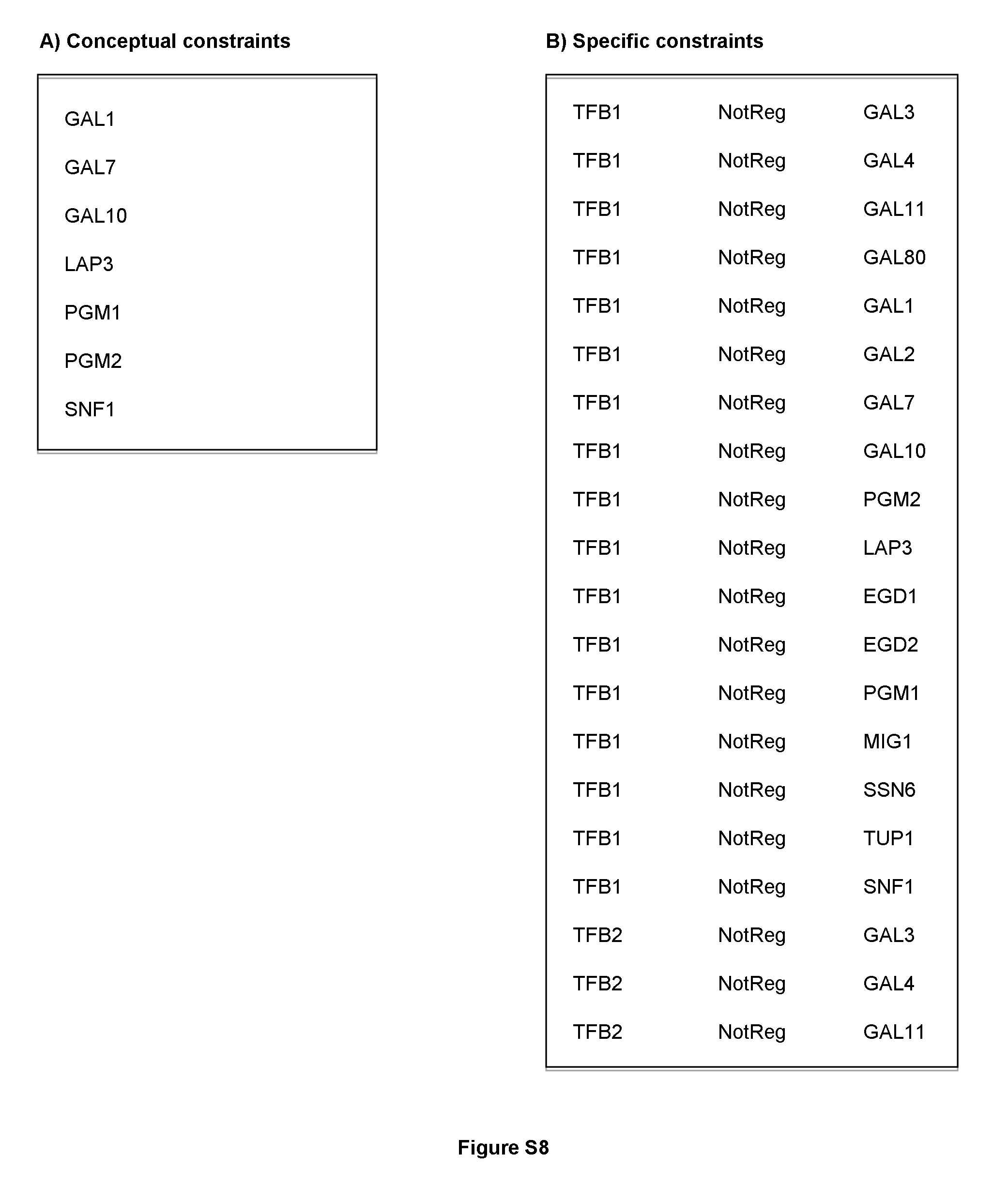

Supplement: Figure S8 — The input file of prior knowledge for constraints-based Boolean network of circadian clock system. The constraints incorporated into the Boolean network can be classified into two types: (A) Conceptual constraints (B) Specific constraints. (TIF) [file pone.0030232.s008.tif]

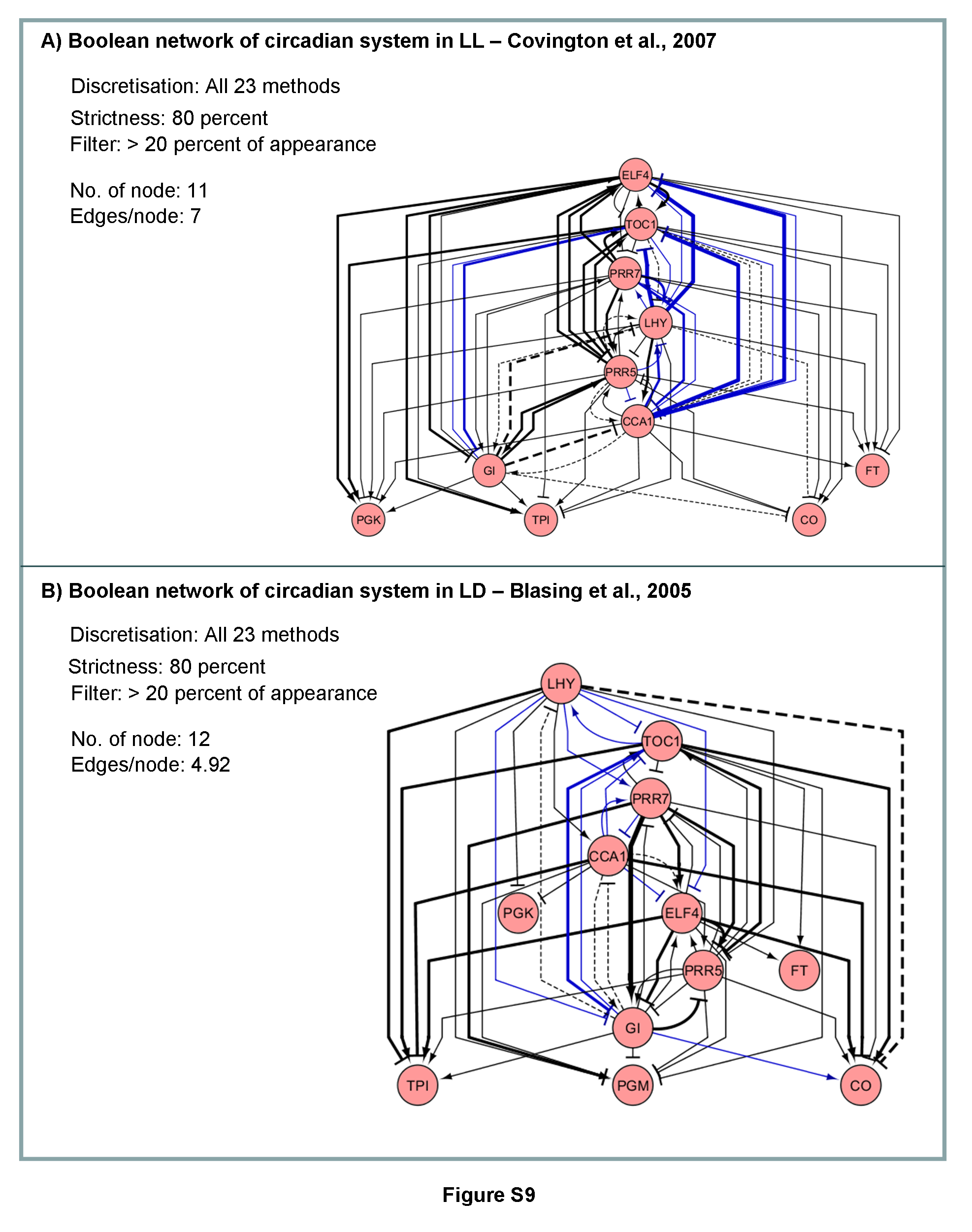

Supplement: Figure S9 — Boolean networks of circadian clock systems. Microarray data measured under different light conditions were analysed by the constraint-based Boolean approach resulting in the inferred networks of circadian clock system (A) under constant light (LL; [14]) and (B) under constant light (LD; [15]). The presented networks were the results of combining 23 individual networks of 23 discretisation methods derived by using level of stringency at 80 percent. (TIF) [file pone.0030232.s009.tif]

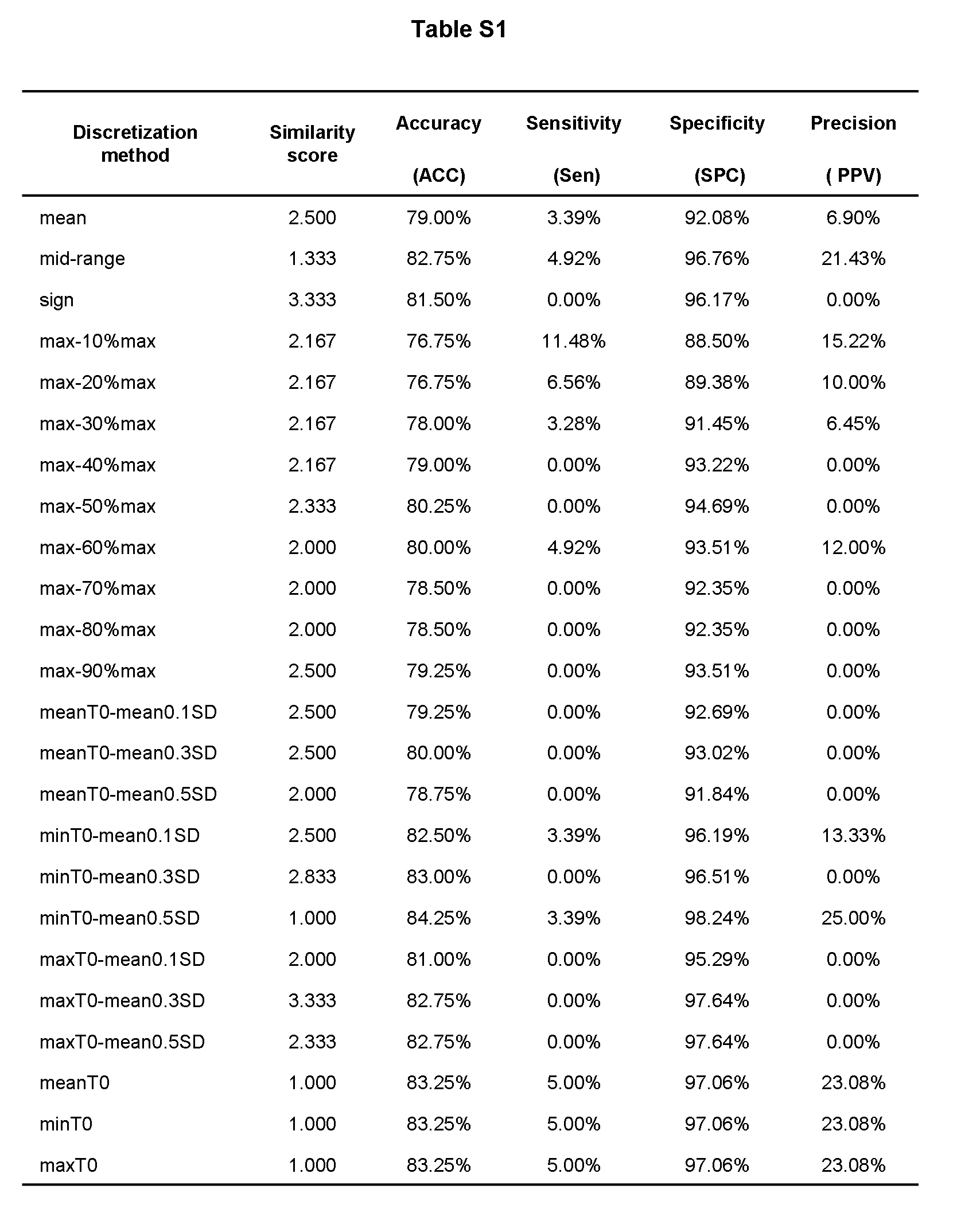

Supplement: Table S1 — Similarity scores of the profiles discretised from various methods. (TIF) [file pone.0030232.s010.tif]
